# Rates and Mechanism of Vivianite Dissolution under Anoxic Conditions

**DOI:** 10.1021/acs.est.3c04474

**Published:** 2023-11-04

**Authors:** Rouven Metz, Naresh Kumar, Walter D. C. Schenkeveld, Stephan M. Kraemer

**Affiliations:** † Centre for Microbiology and Environmental Systems Science, Department for Environmental Geosciences, University of Vienna, Josef-Holaubek-Platz 2, 1090 Vienna, Austria; ‡ Soil Chemistry and Chemical Soil Quality Group, Wageningen University and Research, Droevendaalsesteeg 3, 6708 PB Wageningen, The Netherlands

**Keywords:** stoichiometric dissolution, recycled fertilizer, P recovery, sustainable P cycling, P sink

## Abstract

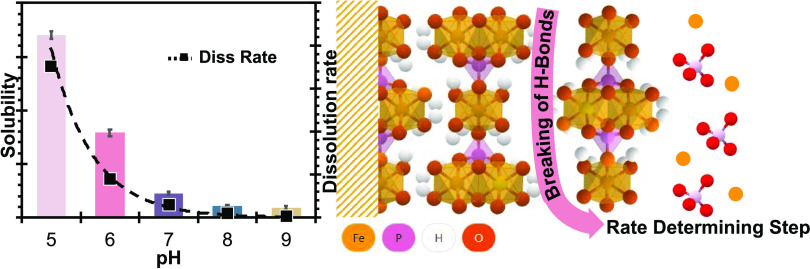

The iron phosphate mineral vivianite Fe(II)_3_(PO_4_)_2_·8H_2_O has emerged as a potential renewable P source. Although the importance of vivianite as a potential P sink in the global P cycle had previously been recognized, a mechanistic understanding of vivianite dissolution at the molecular level, critical to its potential application, is still elusive. The potential of vivianite as a P sink or source in natural or engineered systems is directly dependent on its dissolution kinetics under environmentally relevant conditions. To understand the thermodynamic and kinetic controls on bioavailability, the oxidation and dissolution processes of vivianite must be disentangled. In this study, we conducted controlled batch and flow-through experiments to quantitatively determine the dissolution rates and mechanisms of vivianite under anoxic conditions as a function of pH and temperature. Our results demonstrate that vivianite solubility and dissolution rates strongly decreased with increasing solution pH. Dissolution was nonstoichiometric at alkaline pH (>7). The rapid initial dissolution rate of vivianite is related to the solution saturation state, indicating a thermodynamic rather than a kinetic control. A defect-driven dissolution mechanism is proposed. Dissolution kinetics over pH 5–9 could be described with a rate law with a single rate constant and a reaction order of 0.61 with respect to {H^+^}:  The activation energy of vivianite dissolution proved low (*E*_a_ = 20.3 kJ mol^–1^), suggesting hydrogen bridge dissociation as the rate-determining step.

## Abstract 2

Vivianite is proposed as a recyclable P source, but its behavior in environmental settings is not well studied. This study provides fundamental knowledge on the vivianite dissolution mechanism and kinetics under environmentally relevant conditions.

## 1 Introduction

Phosphorus (P) is an essential element for life and critical for sustaining current agricultural production. While P demand is projected to increase with the growing world population, global P rock reserves are estimated to become depleted within 250–350 years, and supply chain security is further endangered by geopolitical instabilities, classifying P as a critical raw material in the EU (COM/2020/474).^1−3^ Also, excessive fertilizer usage in agriculture during the last century has resulted in elevated inflows of P into water bodies, causing eutrophication.^2,4^ Therefore, new EU (European Green Deal, COM/2019/640) and national regulations (e.g., §3 Absatz 1 AbfKlärV, in Germany) for more efficient P use have been proposed, aiming for a circular economy by recovering and reusing secondary P resources.

The mineral vivianite (Fe_3_(PO_4_)_2_·8H_2_O) has recently gained attention in the context of P recycling. Vivianite is a common Fe(II)-phosphate mineral found globally in marine, freshwater, and terrestrial systems, acting as a substantial P sink.^5−13^ The formation of vivianite is restricted to anoxic, reducing environments at circumneutral pH with locally high concentrations of dissolved PO_4_^3+^ and Fe^2+^, e.g., resulting from microbially mediated organic matter degradation and reductive Fe-(oxyhydr)oxide dissolution. Moreover, vivianite has recently been identified as the main phosphate mineral in sewage sludge of wastewater treatment plants, offering an economically promising P recovery pathway as an alternative to, e.g., struvite ((NH_4_)Mg(PO_4_)·6H_2_O).^14−21^ Pilot studies for efficient vivianite recovery from digested sewage sludge^22−24^ and potential application as a fertilizer^25,26^ have recently been reported. Vivianite is already used as an Fe fertilizer (e.g., in calcareous soils),^27−29^ but it has also been proposed as a potential P fertilizer.^30,31^ However, despite its global significance for the natural P cycle and its technical and agricultural significance, a quantitative and mechanistic understanding of Fe and P mobilization from vivianite is still elusive.^12^

Structurally, vivianite belongs to the *C*2/*m* space group. It consists of sheets comprising single (Fe_I_) FeO_2_(H_2_O)_4_ and double (Fe_II_) Fe_2_O_4_(H_2_O)_4_ octahedral sites that are linked by PO_4_ tetrahedra (the vivianite crystal is visualized in Figure S1). These sheets are only weakly held together by hydrogen bonds between the H_2_O ligands, explaining the perfect cleavage along the 010 plane.^32,33^ Under oxic conditions, vivianite is unstable, and structural Fe(II) oxidizes rapidly to Fe(III), changing the mineral’s appearance from colorless white to bluish and further to deep purple with an increasing degree of oxidation. The oxidation of Fe(II) impacts the crystal structure, as the additional positive charge of Fe(III) needs to be balanced by the release of H^+^.^33,34^ Nevertheless, the vivianite crystal structure seems to be stable until an oxidation degree of ∼50% of total Fe.^32^ Upon further oxidation, vivianite may transform into metavivianite or the amorphous Fe(III)-phosphate mineral santabarbaraite.^35^ Importantly, amorphous Fe(III)-phosphate minerals of various stoichiometry generally have much lower solubilities than vivianite.^36,37^

The bioavailability of Fe and P (from vivianite) is strongly related to their dissolved concentrations, which can be constrained by multiple factors including vivianite solubility, dissolution rates, and mineral transformation reactions. The solubility product of vivianite, defined in eq 1 (braces denote activities), was determined at *K*_sp_ = 10^–36^.^17,38^
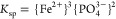
1

Assuming stoichiometric dissolution in the neutral-to-acidic pH range, the dissolved PO_4_^3–^ concentration in equilibrium with vivianite is much higher than that in equilibrium with the Fe(III)-phosphate strengite (FePO_4_·2H_2_O; Figure S2). However, compared to Ca phosphates such as hydroxyapatite (Ca_10_(PO_4_)_6_OH_2_, log(*K*_sp_) = −114)^39^ or common fertilizers, *e.g.*, dicalcium phosphate anhydrous (CaHPO_4_, log(*K*_sp_) = −6.7)^40^ and also compared to struvite (MgNH_4_PO_4_·6H_2_O, log(*K*_sp_) = −13.4),^41^ the expected dissolved PO_4_^3–^ concentrations of vivianite are 1–3 orders of magnitude lower, depending on the pH (Figure S2). In general, bioavailability is not exclusively thermodynamically but often also kinetically controlled; nevertheless, dissolution studies of vivianite are still scarce.^42−44^ Thinnappan et al.^42^ determined a pH-dependent dissolution rate equation for vivianite, with an increasing rate toward basic and acidic pH values and a minimum rate at neutral pH. Protonation and deprotonation of the P–OH group at the vivianite surface result in the weakening of the structural Fe–(PO_4_) bonds due to polarization and promote Fe and PO_4_ detachment, respectively. At basic pH, a preferential release of P was observed, which was attributed to the structural oxidation of Fe. However, the observed dissolution rates for the environmentally relevant pH range of 5–9 were extrapolated from the data only collected for highly acidic and alkaline conditions.^42^ Moreover, oxidation of vivianite was not controlled in any previous study, impeding a thorough interpretation of the results. Even oxidation degrees <50% (within the proposed stability of vivianite) will likely lead to local structural changes in the vivianite crystal and influence its dissolution kinetics. Gypser et al.^43^ and Schütze et al.^44^ synthesized vivianite at an elevated temperature (40 °C) without protection from ambient air, while Thinnappan et al.^42^ obtained a natural vivianite specimen and ground it under atmospheric conditions. However, to gain a quantitative and mechanistic understanding of vivianite dissolution, oxidation and dissolution must be disentangled by ensuring anoxic conditions during the entire experimental procedure, *i.e.*, from mineral synthesis through the actual dissolution experiments.

The aims of the current study were (1) to quantify vivianite dissolution rates under anoxic conditions as a function of pH and temperature, (2) to assess whether thermodynamics or kinetics constrains the availability of Fe and P from vivianite on time scales relevant for application as a fertilizer, and (3) to elucidate the vivianite dissolution mechanism. To this end, a series of controlled batch and flow-through dissolution experiments were performed under strictly anoxic conditions.

## 2 Theoretical Considerations

The experimentally determined surface area normalized dissolution rate (*R*_exp_) in [mol m^–2^ h^–1^] at any time during a batch experiment corresponds to the surface area normalized first derivative of the total dissolved analyte concentration (*C*_diss_) [mol L^–1^] over time (*t*) [h]. Surface area normalization was carried out by dividing the rate in [mol L^–1^ h^–1^] by the specific surface area (SSA) [m^2^ g^–1^] and the suspension density (SD) [g L^–1^]. SD corresponds to the added mass (*m*) [g] of the mineral divided by the suspension volume (*V*) [m^3^] (eq 2)
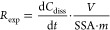
2

In batch experiments, dissolution leads to a continuous increase in dissolved analyte concentration (*C*_diss_) in the reactor until the solid–solution equilibrium is established. Close to equilibrium, the solution saturation state influences *R*_exp_. Therefore, *R*_exp_ cannot simply be equated to the forward dissolution rate (*R*_diss_) of a mineral. However, general rate laws for mineral dissolution have been proposed^45−47^ that include the effect of the solution saturation state on dissolution kinetics (eq 3)
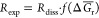
3where *R*_diss_ is the forward dissolution rate per unit surface area of the mineral (mol m^–2^ h^–1^), and  is a unitless function of the molar Gibbs free energy of reaction  (J mol^–1^). The term  accounts for the dependence of the dissolution rate on the solution saturation state. The value of  can be calculated for vivianite by eq 4
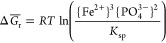
4where *R* is the gas constant (8.314 J K^–1^ mol^–1^), *T* is temperature [K], and *K*_sp_ is the solubility product. For reversible elementary reactions,  can be expressed by eq 5
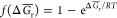
5

Dissolution occurs when ; when , the system is far from equilibrium and  can be approximated to 1.^48^

Equation 5 is only applicable to elementary reactions according to transition state theory. For overall reactions, the function  is difficult to predict.^49^ Empirical adjustments have been made to correct for reactions that include more than one elementary reaction step^49^ by introducing factor *n* into the exponential term such as Kraemer et al.^47^ Furthermore, many minerals show dissolution behavior that is nonlinear in  near equilibrium. To account for this, Berner et al.^50^ successfully included exponential factor *m* to model calcite dissolution. Multiple studies have shown for different minerals that the inclusion of factor *m* is especially useful for reactions that are controlled by crystal defects.^46,49,51−53^ Combining eqs 3 and 5 and including factors *n* and *m* for nonlinear rate laws gives eq 6
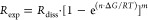
6

For a constant dissolution rate (zero-order), eq 6 can be rewritten as eq 7
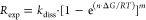
7with the dissolution rate constant *k*_diss_ [mol m^–2^ h^–1^].

## 3 Material and Methods

### 3.1 Materials

All chemical reagents used were ACS grade and used as received. Anoxic water was prepared by bringing ultrapure Milli-Q water (MQ, 18.2 MΩ·cm^–1^, TOC < 2 ppb) to boil and purging it with pure N_2_ (purity ≥ 99.99%) while cooling down (∼4 h). Unless specified otherwise, all experiments and syntheses were performed in anoxic water.

**Figure 1:**
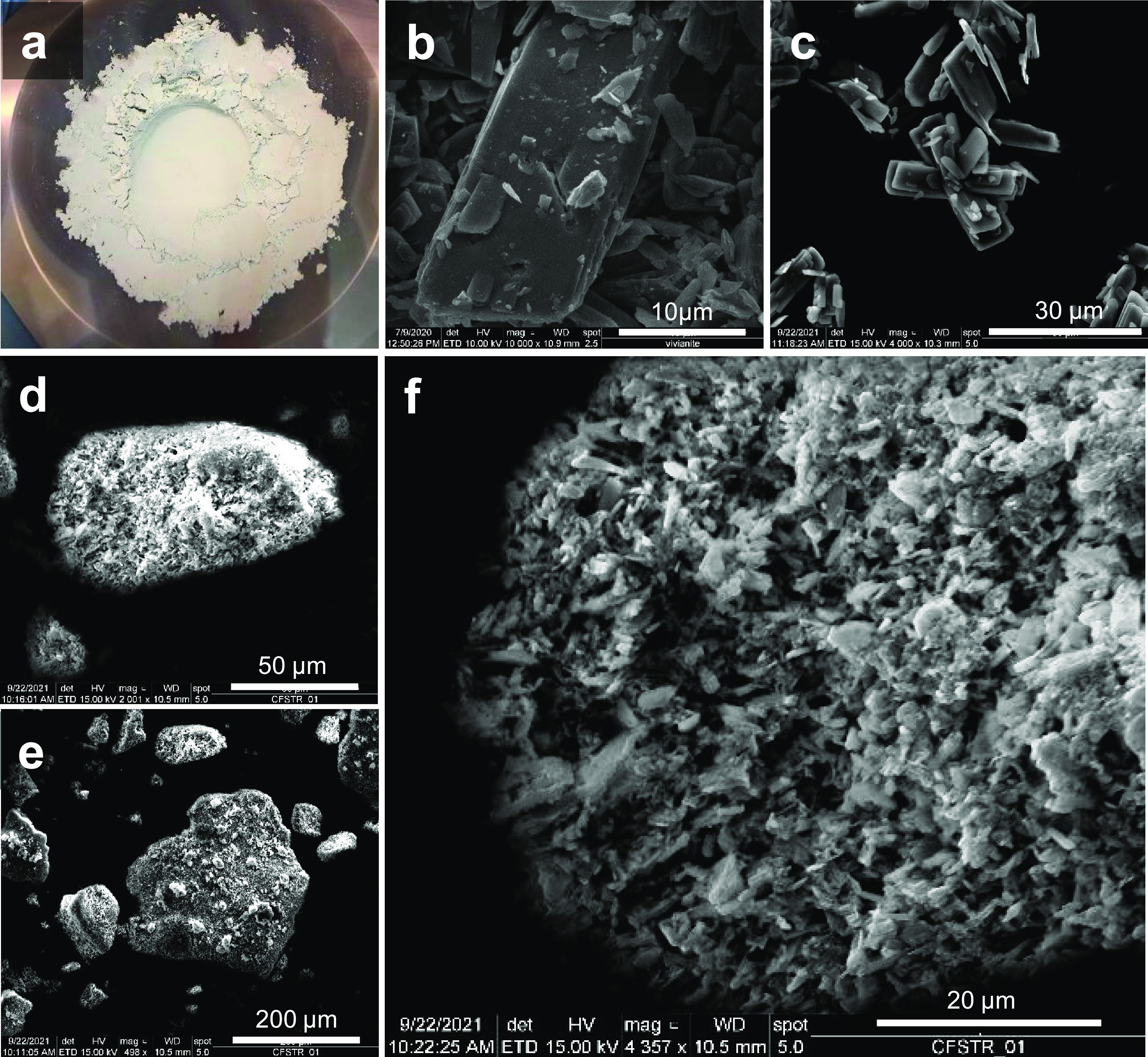
Macro- and microscopic images of synthesized vivianite. (a) Image of synthesized dry vivianite powder. (b) Secondary electron scanning electron microscopy (SEM) image of dry vivianite particles. (c) SEM image of vivianite aged for 14 days in a 100 mM suspension. (d–f) SEM images of agglomerated vivianite particles after ∼30 days of the CFSTR experiment.

Vivianite was synthesized using a modified method of Al-Borno and Tomson^54^ inside an anoxic chamber (mBRAUN, Unilab 7185) under a N_2_ environment (O_2_ < 1 ppm, oxygen analyzer MB-OX-SE-1). Briefly, solutions of 0.4 M NaH_2_PO_4_·H_2_O as the P source and 0.6 M FeCl_2_·4H_2_O as the Fe(II) source were prepared and mixed slowly under constant stirring in a stoichiometric ratio of 2:3 (P/Fe). To induce vivianite precipitation, 0.5 M NaOH was added into the mixed and agitated Fe–P solution with a peristaltic pump at a speed of 10 mL·min^–1^ until the pH increased from 3.5 to 7.0. A white, slightly bluish precipitate formed readily (Figure 1a). The suspension was continuously stirred for ∼24 h, and the pH was adjusted to ∼7 and continuously monitored throughout the synthesis using a pH meter with a glass electrode (Thermo Scientific, Orion 3 star, 9107BN). Finally, the vivianite mineral was collected by vacuum filtration (filter paper, 5–8 μm Whatman) and washed several times with anoxic water to remove excess ions until the electroconductivity was <10 μS cm^–1^. The filtered material was dried at room temperature (20 ± 1 °C), homogenized by a hand pestle and mortar, and stored in a desiccator all inside the N_2_-filled anoxic chamber until used in experiments. To ensure consistency and comparability, all experiments were conducted using vivianite from the same homogenized batch, which was thoroughly characterized before use. Prior to experiments, the dried vivianite was suspended in anoxic MQ to obtain a 100 mM stock suspension.

### 3.2 Experimental Setup

#### 3.2.1 Batch Dissolution Experiment

To investigate the dissolution rate and solubility of the synthesized unoxidized vivianite, batch experiments were performed inside an anoxic chamber at room temperature, using continuously stirred dark brown glass reaction vessels (50 mL) to minimize photo-oxidation. Experiments were initiated by adding 100 μL of a 100 mM vivianite stock suspension to 49.90 mL of a solution containing 15 mM buffer, resulting in 0.2 mM vivianite in the experimental suspensions. Noncomplexing buffers were selected to minimize pH-drift throughout the experiments: *N*,*N*′-diethylpiperazine (DEPP) for pH 5.0 and 9.0, 2-(*N*-morpholino)ethanesulfonic acid (MES) for pH 6.0, 3-(*N*-morpholino)propane-1-sulfonic acid (MOPS) for pH 7.0, and piperazine-1,4-bis(propanesulfonic acid) (PIPPS) for pH 8.0.^55^ The pH was adjusted to the desired value through addition of 1 M HCl or NaOH. The ionic strength (IS) was increased to 10 mM using 1 M NaCl while accounting for the contribution of the buffers. For up to 50 h, samples were collected regularly from the stirred suspensions. One subsample was filtered (0.2 μm CA, Minisart) and stabilized in 1 M HCl and another subsample remained unfiltered and was digested in 6 M HCl. Both filtered and unfiltered samples were analyzed for total dissolved P, Fe, and Fe(II). At the end of the experiment, suspensions were centrifuged, and the remaining solids were collected and stored in the anoxic chamber for solid-phase analysis.

Experiments investigating the effect of temperature on the vivianite dissolution rate were performed in a glovebag (Sigma-Aldrich, AtmosBag), which was continuously flushed with N_2_. The temperature was varied from 5.0 to 75.0 °C and maintained using a thermostat water bath (Huber, KISS k6) and was also monitored with a thermometer placed inside the reaction vessel. The experiments in the glovebag were started after O_2_ levels were below the detection limit (0.1% O_2_, ∼50 ppm) of the oxygen sensor (Greisinger, GOX100). Additionally, the dark brown glass reaction vessels (50 mL) were continuously purged with N_2_ throughout the experiment. Vivianite oxidation was determined by measuring the Fe(II)-to-total Fe ratio at the beginning and the end of the experiment using the Ferrozine method.^56,57^ The water bath with the reaction vessel was placed inside the glovebag on a stirring plate to ensure agitation. The experimental setup and analytical approach were otherwise identical to the batch dissolution experiments described above. All temperature-controlled experiments were conducted at pH 6.0 with 10 mM MES-buffered solution and ran for 2 h at 5.0, 25.0, 50.0, and 75.0 °C. The pH was adjusted at 25 °C, accounting for the temperature dependency of pH to yield pH 6.0 at the desired temperature.

#### 3.2.2 Flow-Through Dissolution Experiment

Continuous flow stirred tank reactors (CFSTRs) with a volume of 90 mL were used to examine the dissolution rate of unoxidized vivianite; a detailed description of the reactor design is presented in Frazier et al.^58^ To prevent O_2_ diffusion over the experimental duration (∼1 month), experiments were conducted inside the anoxic chamber. CFSTRs were wrapped in aluminum foil to prevent photochemical reactions. The reactor outlets were covered by 0.1 μm membrane filters (Whatman, NC10), allowing only effluents to escape the reactor, while the solids were retained inside the reactor. To each reactor, 0.1 g of dried vivianite was added, resulting in a solid-to-solution ratio of 1.11 g L^–1^. Influent solution (Fe and P free, buffered at pH 6 (10 mM MES) with IS = 10 mM (NaCl)) was pumped into the reactor using a peristaltic pump at rates that accommodated a hydraulic residence time between 0.75 and 30 h. Effluent samples (1 mL) were collected at various time intervals, dependent on the pumping rate. Samples were stabilized with 1 M HCl and analyzed for total dissolved Fe and P concentrations. Sporadically, dissolved Fe(II) concentrations were also measured to check for possible Fe oxidation. The remaining effluent solution was used to monitor the pH and the flow rate (gravimetrically). At the end of the experiment, outflow filters with remaining solids were collected and stored in the anoxic chamber for solid-phase analysis.

### 3.3 Analytical Methods

Aqueous samples from batch and flow-through experiments were analyzed for pH and subsequently acidified in a 1:1 ratio with 1 M HCl to stabilize Fe(II) and preserve dissolved species. Solid and suspension samples were completely digested in 6 M HCl. The chemical composition and elemental ratio were determined by measuring total Fe and P concentrations by inductively coupled plasma optical emission spectrometry (ICP-OES; Agilent Technologies, 5110). Fe speciation and the extent of Fe oxidation were determined within 12 h of sampling with the photometric Ferrozine assay,^56^ using the adapted protocol of Porsch and Kappler.^57^ In short, for Fe(II), a buffered Ferrozine solution (0.1% ferrozine, 50% ammonium acetate (w/v)) was added to dissolved and acidified samples. For Fe(tot), before adding Ferrozine solution, 10% (w/v) hydroxylamine in 1 M HCl was added to the sample. Absorbance was measured at 562 nm with a UV–vis spectrophotometer (Tecan, Infinite M Plex). The degree of oxidation was calculated using eq 8
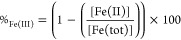
8

Dissolved total PO_4_ concentrations were determined by an adapted molybdenum blue method,^59^ where ammonium molybdate and potassium antimonyl tartrate react in an acidic medium with orthophosphate to form phosphomolybdic acid, which is subsequently reduced by ascorbic acid to molybdenum blue. Absorbance was measured spectrophotometrically at 880 nm (Tecan, Infinite M Plex).

The phase purity and secondary mineral formation were verified by X-ray powder diffraction (XRD) analysis (Rigaku, Miniflex 600 equipped with Cu Kα radiation (λ = 1.54 Å) and a monochromator) with 2θ from 10 to 80° with step sizes of 0.06° and 0.5° min^–1^ using an anoxic sample holder with a zero-diffraction silicon base. Mean particle size was determined by laser obscuration (ambivalue, EyeTech Combi). The specific surface area was determined through BET analysis (Quantachrome, Nova 2000e) by N_2_ adsorption at room temperature and degassing under vacuum overnight. Scanning electron microscopy (SEM; FEI, Inspect S50) equipped with an energy-dispersive X-ray detector (EDX, Apollo XV) was used to examine the particle sizes and morphology of the solid material. Ethanol-suspended samples were deposited on sample holders with double-sided tape and carbon coating (Leica, EM SCD 500) before SEM analysis. Image analysis for particle size measurement was performed using Fiji software.^60^ During the sample preparation for BET and SEM, the exposure to oxygen could not be completely prevented, but exposure time during weighing, sputter coating, and sample introduction was minimized.

### 3.4 Equilibrium Calculations

Calculations of equilibrium speciation were performed using VisualMINTEQ 3.1 and its associated thermodynamic database (thermo.vdb). Measured total dissolved Fe and P concentrations, along with other solution composition parameters (*e.g.*, pH and electrolyte concentration), were used to model species activities for calculating solubility products. Reducing conditions were simulated by fixing pe at –4. The pH was fixed to the measured value, while IS was calculated from electrolyte concentrations in the modeled solutions. The modeled activities were used to calculate  values according to eq 4.

## 4 Results

### 4.1 Properties of Synthetic Vivianite

The synthesized vivianite had a white, slightly bluish color (Figure 1a), and XRD analysis confirmed a pure crystalline phase (Figure S3). The BET specific surface area (SSA: 1.24 m^2^ g^–1^) was within the range of previously reported values of 0.2–4.8 m^2^ g^–1^.^38,42^ The particle size distribution (number based) measured by laser obscuration gave a mean diameter of 9.5 ± 6.8 μm (D10: 2.85 μm; median/D50: 7.87 μm; D90: 17.6 μm), which reflects crystal sizes previously found in digested sludge.^22^ The P/Fe ratio in the acid digest was ∼0.67, in accordance with the mineral stoichiometry. The determined percentage of oxidation (eq 8) was <1%. In the SEM images of the initial dry vivianite powder (Figure 1b), many small particles appeared to be attached to the elongated larger crystals, which had many surface defects such as cracks, holes, and shifts in stacked sheet layers. In the vivianite stock suspension, a ripening of the crystals was observed, leading to fewer small particles and defects due to dissolution at reactive sites and crystal growth (Figure 1c). The composition of crystals was semiquantitatively assessed by EDX measurements, and the elemental ratio of P/Fe did not differ from the value of the acid digest.

### 4.2 Solubility of Vivianite

**Figure 2:**
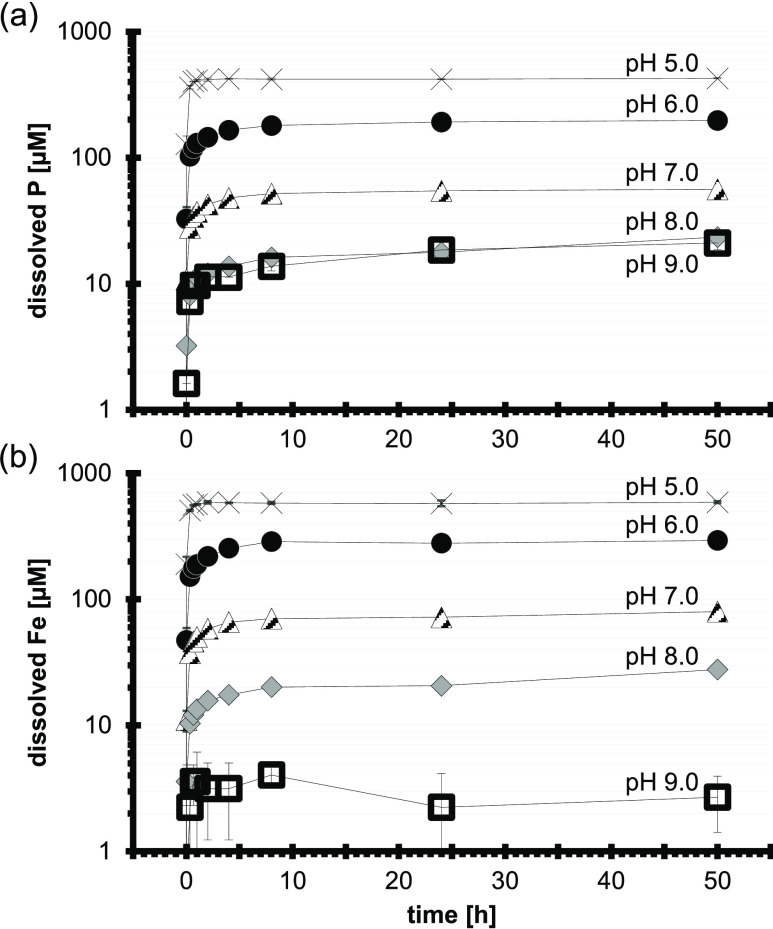
Dissolution of vivianite (200 μM) over an environmentally relevant pH range (5–9) under anoxic conditions in a buffered solution (IS = 10 mM). (a) dissolved PO_4_ and (b) Fe concentrations over time. Error bars indicate deviations between duplicates.

Figure 2 shows the results from batch dissolution experiments with 200 μM vivianite at various pH values. The dissolved P and Fe concentrations increased rapidly within the first 2 h and thereafter remained mostly steady for the duration of the experiments (50 h). The dissolved concentrations at the steady state increased strongly with decreasing pH: almost a 100-fold increase from the highest (pH 9) to the lowest (pH 5; Figure 2). At pH 5, all solid material had completely dissolved during the fast initial dissolution. For the remaining pH values, this was not the case, and equilibrium was assumed to be reached. From the total dissolved Fe and P concentrations at 50 h, the Fe^2+^ and PO_4_^3–^ activities were determined by speciation modeling. These were used to calculate the *K*_sp_ according to eq 1. The average calculated p*K*_sp_ for the pH range of 6–9 was 33.6 (±0.5). All calculated *K*_sp_ values for vivianite were within the range of previously reported values, which is relatively broad (29.9–36;^38,54,61,62^Figure S4).

### 4.3 Vivianite Dissolution Kinetics

During the course of the CFSTR experiments, the measured P and Fe effluent concentrations decreased slowly but constantly over time despite changes in the flow rate (Figure S5). At feasible pumping rates, the dissolution kinetics of vivianite were fast relative to the average residence time of the solution inside the reactor. Therefore, effluent concentrations and the corresponding calculated dissolution rates were controlled by equilibrium with vivianite and the flow rate, respectively. Initially, the effluent concentrations of the CFSTR experiments were comparable to equilibrium concentrations determined in batch experiments. A substantial fraction of the initial vivianite dissolved during the experiment (>50%), resulting in a decrease in mineral surface area in suspension. Additionally, SEM images of the solids remaining at the end of the experiment show strong agglomeration of the smaller vivianite particles (Figure 1d–f). Both effects can contribute to a decreasing *R*_exp_ over a longer time period, even at a constant solution residence time. The gradual decrease in effluent concentrations may result either from a decline in vivianite solubility or from a transition from effluent concentrations being controlled by solubility to a control by (declining) dissolution rates. A detailed discussion of the CFSTR experiment is presented in the SI.

**Figure 3:**
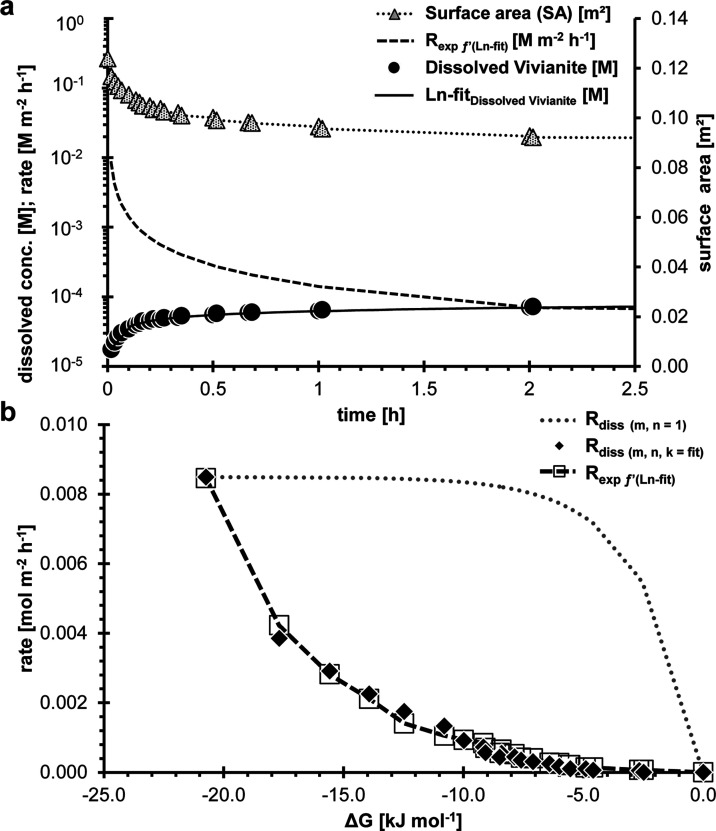
(a) Observed dissolved concentration of vivianite (filled circles) during dissolution under anoxic conditions at pH 6.0 (10 mM MES; *I* = 10 mM); calculated from the dissolved Fe and P concentration, assuming Fe_3_(PO_4_)_2_·8H_2_O stoichiometry. The black solid line visualizes a logarithmic function fitting the observed vivianite dissolution. Gray triangles show the decrease in surface area over time. The black dashed line represents the first derivative of the logarithmic fit of the surface area normalized vivianite dissolution data (*R*_exp_). (b) Surface area normalized dissolution rate at pH 6.0 as a function of the Gibbs free energy of reaction (Δ*G*). The first derivative of the logarithmic fit of the dissolved vivianite concentration (*R*_exp_) at sampling points (open squares), and dissolution rate (*R*_diss_) according to eq 6 with *n* = 0.2 and m = 4.7 (R^2^ = 0.96); Δ*G* was calculated for the individual sampling points by eq 5 (filled squares). Thus, the vivianite dissolution rate can be related to the experimentally determined solution saturation state. For comparison, the rate calculated with a linear rate law according to transition state theory is presented (*R*_diss_ with n, m = 1; dashed/dotted gray line).

In order to resolve the fast initial vivianite dissolution kinetics for the stage where the solution is still far from equilibrium and kinetics is thus not significantly influenced by approaching equilibrium conditions, a separate batch experiment was performed. Concentration data from this experiment were used to parametrize a rate law equation and to determine a rate constant. The batch experiment ran for 50 h, and no agglomeration was observed (Figure S7d–f). Therefore, possible effects of agglomeration on the reactive surface area and observed dissolution rates were not further investigated in this study. The last data point was used as an estimate for the equilibrium concentration (Δ*G* = 0). Figure 3a shows the dissolved vivianite concentration, calculated from dissolved Fe and P concentrations, as a function of time in a batch experiment with a 200 μM vivianite suspension (pH 6.0, IS = 10 mM). During the very fast initial dissolution, samples were taken every minute. As the system approached equilibrium, the dissolution rate rapidly declined. Due to substantial dissolution (∼50% of the initial material), the reactive surface area could not be considered constant. Therefore, a mass-based surface area correction was applied using eq 9, which was developed for a similar problem in calcium phosphate dissolution^63^
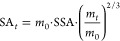
9where SA_*t*_ is the surface area at time *t*, *m*_0_ is the initial mass of added vivianite, SSA is the determined specific surface area of the starting material, and *m*_*t*_ is the mass at time *t*. The value for *m*_*t*_ was calculated from the difference between the total suspension concentration and the dissolved concentration of vivianite. The dissolved vivianite concentration was calculated from the dissolved Fe(tot) and P concentrations, accounting for the mineral stoichiometry. SA_*t*_ was calculated for each sampling point and used for surface normalization. The dissolved vivianite concentrations were fitted to a logarithmic function (Figure 3a). The first derivative of the logarithmic fit describes the observed dissolution rate *R*_exp_.

*R*_exp_ was then used to fit *R*_diss_ according to eq 6. While *m* and *n* are considered fitting parameters, *k*_diss_ can be estimated by the slope of the linear part of the initial dissolution reaction (eq 7, Figure 3a). However, vivianite dissolution kinetics was very fast, and only the first sampling points could be included to determine the initial rate (where *R*_exp_ ≈ *R*_diss_), contributing to uncertainty in the estimated value for *k*_diss_. In order to test the validity of this approach, we also fitted the data with *k*_diss_ as an additional fitting parameter. This, however, did not improve the quality of the fit significantly, yet the fitted and estimated values of *k*_diss_ were in good agreement (*k*_diss_ estimated: 8.4 × 10^–3^; fitted: 8.5 × 10^–3^ in mol m^–2^ h^–1^), thus supporting each other. The values for the fitting parameters *n* and *m* were 0.2 and 4.7, respectively.

#### 4.3.1 Dissolution Rate over the Experimental pH Range

Apart from the solution saturation state, the pH also had a strong influence on the dissolution rate of vivianite (Figure 2). At high pH values (pH 8 and 9), vivianite changed visibly to a slightly reddish color, suggesting the potential formation of Fe(III) precipitates (Figure S6, suspension oxidation degree). Even though no changes in the XRD patterns were observed for the material (Figure S3), amorphous surface precipitation cannot be completely ruled out. Elemental analysis following acid digestion of the vivianite stock suspension (100 mM vivianite suspended in unbuffered MQ) indicated that the molar ratio of P/Fe was 0.67 ± 0.02, which corresponds to the stoichiometric ratio of initial vivianite. Microscopic examination of the remaining solids did not reveal significant morphological changes (Figure S7c–f). Also, no changes in the P/Fe ratio between bulk and edge particle regions were detected using elemental mapping with EDX. The Fe and P solution concentrations from the dissolution experiments were used to examine if dissolution was congruent. For the pH range between 5 and 7, this was indeed the case. For pH 8 and 9, however, a preferential release of P was observed, and the dissolved P/Fe ratio increased strongly with pH (pH 8: 0.8; pH 9: 7.0). So, especially at pH 9, the formation of secondary amorphous Fe(III) precipitates could not be excluded. Therefore, net dissolution rates as a function of pH were calculated only from dissolved P concentrations and normalized to the vivianite stoichiometry. Adsorption of P to Fe(III) precipitates was assumed negligible due to low Fe(III) concentrations measured in suspension (Figure S6) and the high P/Fe ratio^64^ in solution, suggesting that the loss of P due to adsorption was marginal in our experiments.

**Figure 4:**
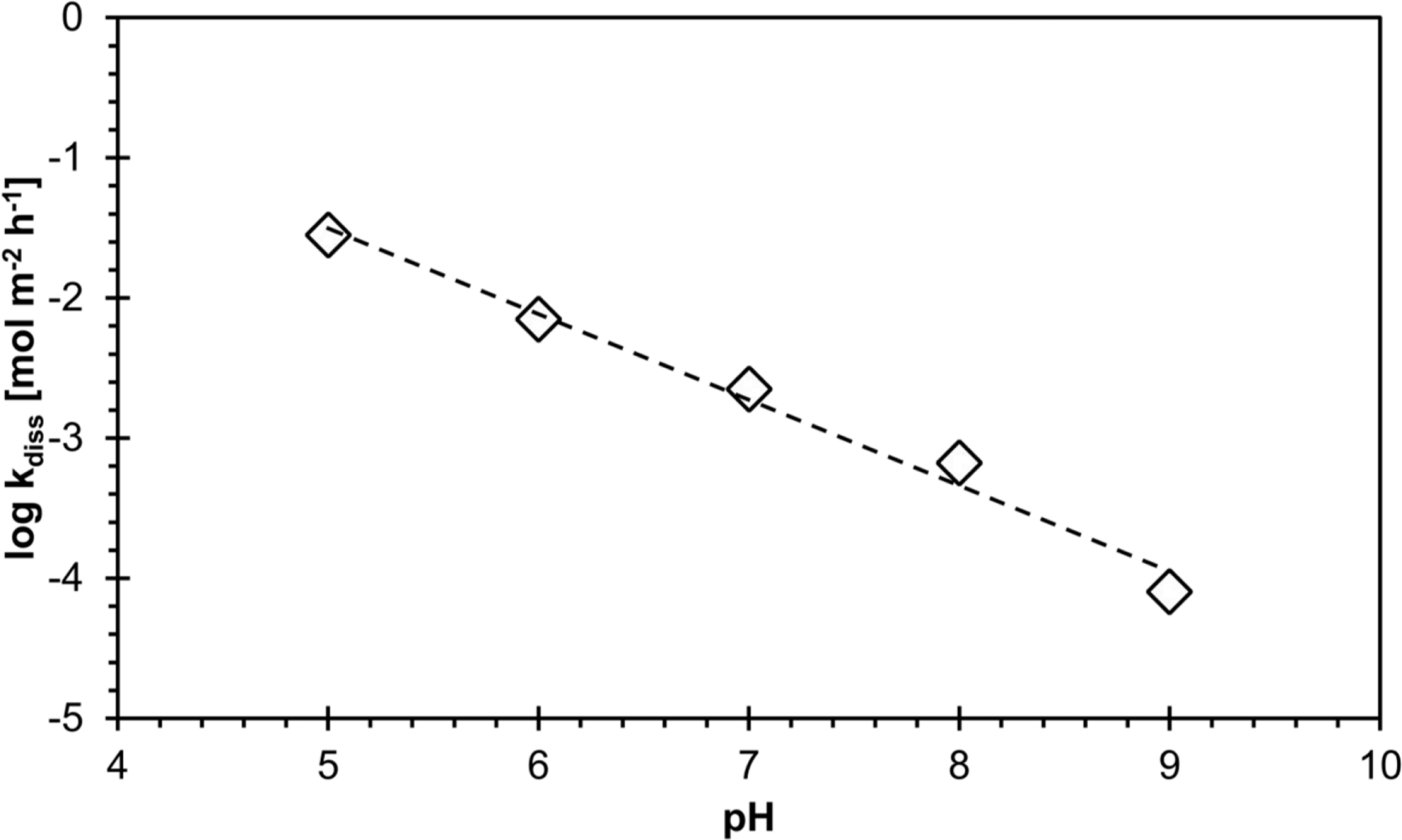
Decadic logarithm of the dissolution rate constant *k*_diss_ (mol m^–2^ h^–1^) of vivianite as a function of pH. Reaction conditions: anoxic, 200 μM vivianite in a solution containing 10 mM pH buffer (IS = 10 mM, NaCl).

An equation to describe the pH dependence of the dissolution rate constant of vivianite (under far-from-equilibrium conditions; *k*_diss_) was derived. Initial dissolution rates were determined from the change in concentration between *t =* 0 and the first sampling point (*t* = 1 min), since estimation through linearization was in good agreement with fitting *k*_diss_, as previously shown. The dissolution rate constants (*k*_diss_) for the various pH values were calculated using eq 7 (with Δ*G* ≪ 0). The logarithm of the rate constant proved to decrease linearly with an increasing pH (Figure 4; eq 10). Or, in other words, the rate constant increased linearly with the proton solution concentration to the power of reaction order *z* (eq 11). *k*_0_ (mol^1–*n*^ m^–2^ h^–1^) represents the rate constant at pH 0.
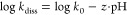
10
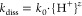
11

When applied to the rates measured for pH 5–9, linear regression to eq 10 (*R*^2^ = 0.99; Figure 4) yielded a reaction order *z* of 0.61 ± 0.04 and a value for rate constant *k*_0_ of 36.0 ± 1.28 mol m^–2^ h^–1^ (log *k*_0_*=* 1.56 ± 0.11).

Using the fitted parameters to express *k*_diss_ in terms of pH, we arrived at the following expression, eq 12
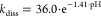
12

The combination of eq 12 with the solution saturation-dependent dissolution rate (eq 7) resulted in an expression for the overall vivianite dissolution rate (*R*_exp_), eq 13
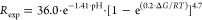
13

#### 4.3.2 Temperature Dependence of the Vivianite Dissolution Rate

**Figure 5:**
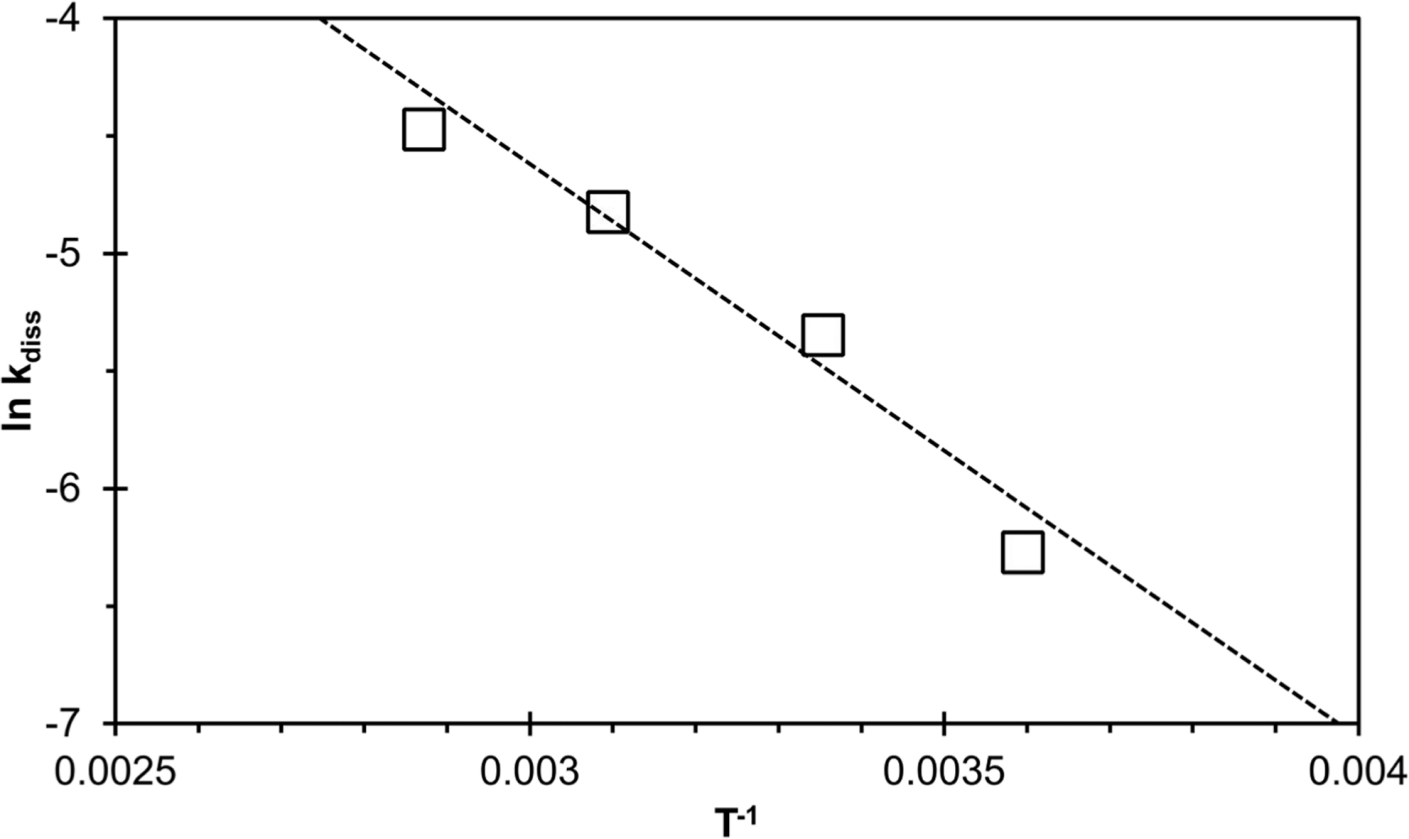
Arrhenius plot for pure vivianite at pH 6.0 (MES-buffered). The dissolution rate constant *k*_diss_ is expressed in mol m^–2^ h^–1^, and the temperature ranged from 5 to 75 °C. *E*_a_ was calculated from the slope of the linear fit and equaled to 20.3 ± 3.0 kJ mol^–1^ (IS = 10 mM, NaCl).

Temperature (5–75 °C) dissolution experiments were performed in order to determine the activation energy of the rate-limiting step of the dissolution reaction. The activation energy can help us to further understand vivianite dissolution and its high dissolution rates. Initial vivianite dissolution rates increased with temperature from 1.9 × 10^–3^ (5 °C) to 1.1 × 10^–2^ (75 °C) mol m^–2^ h^–1^ (Figure S8). The natural logarithms of the normalized dissolution rate constants at pH 6, determined far-from-equilibrium conditions, were plotted against the reciprocal temperature, resulting in an Arrhenius plot (Figure 5). The activation energy was calculated using the Arrhenius equation (eq 14)
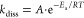
14where *A* is referred to as the pre-exponential factor, *E*_a_ is the activation energy, *R* is the gas constant, *T* is the temperature in degrees Kelvin, and *k*_diss_ is the rate constant. The slope of the Arrhenius plot corresponds to −*E*_a_/*R*, resulting in *E*_a_ = 20.3 ± 3.0 kJ mol^–1^.

## 5 Discussion

### 5.1 Solubility Product

The variation in reported solubility products among studies (Figure S4^38,54,61,62^) cannot be explained by the temperature dependence of *K*_sp_, since it is negligible for vivianite; *K*_sp_ increases by only 0.14 log units from 5 to 45 °C.^54^ Instead, variations might result from differences in the particle size and degree of vivianite crystallinity. The apparent solubility product (*K*_sp(S)_) for small particles, especially those smaller than 1 μm or with specific surface areas greater than a few m^2^ g^–1^, can be estimated from the solubility product for infinitely large particles (*K*_sp(S→0)_), the molar surface *S* [m^2^ mol^–1^], and the mean Gibbs free energy of the solid–liquid interface γ̅ [J m^–2^]^39^ (eq 15).
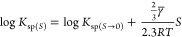
15

Pinto et al.^65^ computed the γ̅ of the (010) vivianite face to be 0.23 J m^–2^. Additionally, γ̅ was estimated according to the method described by Schindler^66^ with 0.31 ± 0.03 J m^–2^. Taking this surface free energy, the apparent solubility would only substantially increase above a molar surface area of 10000 m^2^ mol^–1^, which is approximately 16-fold higher than the measured surface area in this study (*S*_vivianite_ ∼620 m^2^ mol^–1^; Figure S9).

However, observed *K*_sp(S)_ values were also reported to vary with pH within individual studies that consistently used the same starting material. Both in the present study and in Singer,^61^ the solubility product appeared to increase with increasing pH until pH > 8, where nonstoichiometric dissolution complicates the calculation of *K*_sp(S)_. Since *K*_sp(S)_ values are by definition pH-independent, first, several databases (phreeqc.dat, minteq dat, minteq v4.dat) were reviewed to ensure correct species distribution calculations and consistency of constants. Second, protons were included in the *K*_sp(S)_ equation. Under the constraints of charge balance and a constant *K*_sp(S)_ value within the pH range of 6–8, we optimized the stoichiometric coefficients for Fe^2+^ and H^+^ in the mineral structure and *K*_sp(S)_ by minimizing the deviation of the modeled from the measured dissolved Fe^2+^, PO_4_^3–^, and H^+^ concentrations (a more detailed description can be found in the SI, eq S3 and Table S1). Deviations were minimal, and the optimization yielded the following solubility (eq 16)
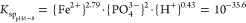
16implying a P/Fe ratio of 0.72. This result contrasts with the 0.67 ± 0.02 ratio determined by complete acid digestion. Possibly, crystal defects at the vivianite surface (*e.g.*, vacancies) lead to a locally somewhat different surface stoichiometry compared to the bulk. With increasing pH, less vivianite dissolved, and defects may have had a more prominent effect on the overall stoichiometry of dissolved species. Further measurements are required to verify this hypothesis. Including a proton term in the solubility product would also reduce the pH-related variability in *K*_sp_ values previously reported for vivianite (Figure S4). Throughout the remainder of this study, vivianite stoichiometry was assumed to correspond with the bulk (P/Fe ratio = 0.67).

### 5.2 Dissolution Rate and pH Dependence

The determined dissolution rate for vivianite (8.5 × 10^–3^ mol m^–2^ h^–1^ at pH 6.0, and 8.0 × 10^–5^ mol m^–2^ h^–1^ at pH 9.0) is much higher than that for Fe(III) oxides (*e.g.*, goethite 10^–9^ mol m^–2^ h^–1^ at pH 5.0^67^). This can be explained by the larger bond strength of Fe(III)–O bonds than that of Fe(II)–O bonds, with a smaller Madelung energy, and further by the more dynamic nature of the Fe(II) coordination sphere than that of the Fe(III) coordination sphere.^68^ Therefore, reductive dissolution is a common strategy for organisms to increase Fe bioavailability.^69−71^ However, the determined vivianite dissolution rate is also high compared to reported values for other common phosphate minerals in the environmentally relevant pH range at 25 °C, *e.g.*, ∼2 × 10^–7^ mol m^–2^ h^–1^ for hydroxyapatite (Ca_10_(PO_4_)_6_(OH)_2_) at pH 5.9–8.45;^72^ ∼5.7 × 10^–8^ mol m^–2^ h^–1^ for fluorapatite (Ca_5_(PO_4_)_3_F) at pH ∼ 9.7;^73^ ∼2 × 10^–8^ mol m^–2^ h^–1^ for variscite (AlPO_4_·2H_2_O) at pH 8.99;^74,75^ and ∼5 × 10^–6^ mol m^–2^ h^–1^ for struvite (MgNH_4_PO_4_·6H_2_O) at pH 8–11.^74^ Roncal-Herrero and Oelkers^74^ stated that fast struvite dissolution was facilitated by the presence of H-bonds in the structure. H-bonds are relatively weak compared to ionic and covalent bonds and are absent in the structure of most other phosphate phases. Also, in vivianite, weak H-bonds are present along the 010 plane, sustaining the layered structure. However, while struvite dissolution rates are pH-independent at pH 7.5–11,^74^ vivianite dissolution rates increase strongly with decreasing pH. The strong increase in solubility and dissolution rate of vivianite with decreasing pH might be explained by proton-promoted dissolution, as suggested for metal oxides.^76−79^ Proton binding weakens critical Fe–O bonds with the underlying lattice by electrophilically attacking metal–oxygen–metal bonds, thus polarizing and imparting surface charge. The proton thus changes the coordination environment of Fe and facilitates its release from the oxide surface.^80^ Similarly, Bengtsson and Sjöberg^81^ demonstrated the formation of protonated phosphate-containing surface sites for apatite, resulting in a proton-promoted dissolution. The most stable surface of vivianite is the (010) plane terminated by Fe octahedra with H_2_O ligands (Figure S1).^65^ Depending on the solution pH, these H_2_O ligands are protonated, which promotes the dissolution of the underlying Fe–PO_4_ sheet, which, in turn, is only weakly connected *via* hydrogen bonds to the next layer.^82^

Alternatively, particularly at above neutral pH, alkaline dissolution involving deprotonation of surface groups as a precursor step to dissolution can occur as observed, *e.g.*, by Blum and Lasaga^83^ for albite. However, for vivianite, a decreasing dissolution rate was observed even above neutral pH, and therefore, alkaline dissolution is considered unimportant within the examined pH range.

The observed nonstoichiometric dissolution at alkaline pH (>7), characterized by the preferential release of P, might result from (a limited extent of) Fe oxidation and Fe(III) precipitation, as argued by Thinnappan et al.^42^ Further, Senn et al.^84^ determined a maximum P/Fe ratio of ∼0.7 in precipitates formed during Fe(II) oxidation in the presence of dissolved P, which is close to the stoichiometric P/Fe ratio of vivianite (0.67). Thus, vivianite oxidation might generate amorphous Fe(III)-phosphate precipitates that could incorporate all released PO_4_. Subsequently, aging effects could lead to transformations of these secondary Fe(III)-phosphate precipitates and are associated with a loss of oxyanion retention capacity, resulting in the release of initially coprecipitated P.^85^ However, the short runtime (50 h) of our dissolution experiments does not support aging as the main cause for preferential P release. Moreover, the measured data does not reflect the oxidation of Fe to a substantial degree (Figure S6). Alternatively, amorphous Fe(II)(OH)_2_ may have precipitated at pH 9, shifting the elemental ratio of dissolved P/Fe. Stoichiometric dissolution up to the measured P concentration would have resulted in oversaturation of Fe(II)(OH)_2_.

### 5.3 Dissolution Mechanism

Calculations of dissolution rates were based on the observed initial dissolution, since the observed rates decreased when equilibrium was approached. Nonlinear rate laws as a function of solution saturation state are often considered to describe reactions controlled by crystal defects.^46,79^ They typically involve exponential factor *m* (eq 6) and have previously successfully been used to describe the dissolution kinetics for, *e.g.*, kaolinite,^53,86^ gibbsite,^49^ quartz,^51^ and analcime.^52^ Previous studies^87−89^ tried to relate the values of *m* to dissolution mechanisms. However, the attribution of a dissolution mechanism based solely on the value of exponent *m* cannot be justified and requires further validation.^79^

In this context, it is important to note that the SEM images of the vivianite used in our study showed clearly visible defects on crystal surfaces (Figure 1b). This suggests that the dissolution rate of vivianite as a function of Δ*G* may indeed be related to crystal defects (Figure 3b), as discussed above.

The SEM images of the reacted vivianite show fewer defects and more rounded shapes than those of the initial dry powder. This indicates that dissolution had approached equilibrium, where nucleation of pits is not energetically favored, and dissolution occurs rather as steps sweep across the crystal surface with new steps only nucleating at crystal edges. Consequently, surfaces become smoother rather than more pitted, and edges become rounded,^79^ corresponding with our observations (Figures 1b,c and S7b,c) and supporting the use of eq 6.

The activation energy (*E*_a_) we determined for vivianite dissolution was only 20.3 ± 3.0 kJ mol^–1^. This is lower than the reported values for struvite, 37.6–44.8 kJ mol^–1^,^90^ but higher than that for the commercial P fertilizer single superphosphate (Ca(H_2_PO_4_)_2_): 14.3 kJ mol^–1^.^91^ In general, mineral dissolution reactions with an activation energy as low as ∼20 kJ mol^–1^ have been interpreted as rate-limited by diffusion of reaction products away from the mineral.^67,79^ The low *E*_a_ also suggests that the breaking of hydrogen bonds might be the rate-determining step in vivianite dissolution, similarly as proposed for struvite dissolution.^74^ However, vivianite has a layered structure where weak hydrogen bonds between the H_2_O ligands of Fe(II) octahedra hold together sheets consisting of ionically bound Fe(II) octahedra and PO_4_ tetrahedra.^92^ Accordingly, vivianite dissolution along this weak (010) surface plane might accelerate the dissolution rates. In this sense, a mechanism similar to exfoliation is hypothesized, where fragments of Fe–PO_4_ sheets peel off the mineral along the (101) plane (Figure S1).^93^

The proposed mechanism of defect-driven dissolution of vivianite corresponds to the fitted rate eq (eq 6), with exponential factor *m* accounting for defect-controlled dissolution, and is supported by SEM observations. The weakly bound stacked sheets lead to high-energy surface sites ranging from adatoms to step retreats, which control the dissolution rate. Naturally grown vivianite crystals are formed more gradually than freshly precipitated vivianite synthesized at high supersaturation. Therefore, natural specimens contain fewer crystal defects and high-energy sites. In addition to oxidation and partial isomorphous substitution, this lack of high-energy sites may slow down the dissolution kinetics of vivianite. Still, the use of ground natural vivianite specimens is not preferred because grinding is hypothesized to induce an autoreduction–oxidation process initiated by rupturing hydrogen bonds during cleavage of (010) surfaces.^94^

## 6 Environmental Implications

Vivianite offers a promising way for recovering P from secondary sources such as wastewater sewage sludge, since the efficiency and feasibility of the recovery process have already been proven.^19,23,95^ The success of vivianite as a recycled P source depends not only on its recovery but also on its reusability, *e.g.*, as a fertilizer. A quantitative understanding of dissolution rates and mechanisms is fundamental to understanding the suitability of vivianite as a P source. The determined dissolution rates of vivianite are fast and exceed the dissolution rates of other common phosphate minerals such as struvite, variscite, or apatite, potentially contributing to its efficiency as a fertilizer. Commercially available P fertilizers combine high dissolution rates with high solubilities, which result in a pulsed P release into a soil solution. In laboratory experiments, pristine vivianite showed sufficiently high solubility over the entire pH range tested (5–9) to support optimal plant growth (1–60 μM P).^96^ However, in natural soils, vivianite oxidation and the presence of alternative P sinks may impact the solubility. Furthermore, aging effects and especially oxidation might substantially change the properties of vivianite and, consequently, the extent to which P from vivianite becomes bioavailable in soils. Therefore, alkaline calcareous soils may be disadvantageous for vivianite application due to the lower dissolution and faster oxidation kinetics of dissolved Fe(II). The effectiveness of vivianite as a P fertilizer is co-determined by a variety of biogeochemical and hydrological soil processes and conditions. Key processes and conditions include the water flow rate through the soil, the presence of organic ligands that form stable complexes with Fe, the presence of reactive clay and (oxyhydr)oxide mineral surfaces that adsorb P, the uptake of P by soil organisms, etc. The mechanistic understanding of anaerobic vivianite dissolution discussed in this study can serve as a basis for investigating the aforementioned processes in natural settings. Also, since vivianite is metastable in oxic environments and most agricultural soils are oxic, the impact of oxidation on the dissolution kinetics and solubility of vivianite is of particular interest; this issue will be addressed in a follow-up study.

## Supporting Information Available

Vivianite crystal structure; equilibrium concentration of dissolved P of vivianite in comparison to other phosphate minerals; XRD diffractograms; solubility product of vivianite in comparison to previous studies; results and discussion of a flow-through dissolution experiment; oxidation degree of vivianite suspensions; SEM images of vivianite before and after dissolution experiments; temperature-dependent dissolution kinetics; solubility product as a function of molar surface area; and optimization of vivianite stoichiometry with respect to *K*_sp_ (PDF)
