# Supplementary material for: Rates and Mechanism of Vivianite Dissolution under Anoxic Conditions
Source: Environ Sci Technol. 2023 Nov 4;57(45):17266–77. doi: 10.1021/acs.est.3c04474 (PMC10653223; doi:10.1021/acs.est.3c04474)
Supplement: Supplementary file 1 — es3c04474_si_001.pdf [file es3c04474_si_001.pdf]

Rates and Mechanism of Vivianite Dissolution under anoxic conditions

Rouven Metz[a], Naresh Kumar[b]*, Walter D.C. Schenkeveld[b]* and Stephan M. Kraemer[a]

[a] Centre for Microbiology and Environmental Systems Science, Department for Environmental Geosciences, University of Vienna, Josef-Holaubek-Platz 2, 1090 Vienna, Austria

[b] Soil Chemistry and Chemical Soil Quality Group, Wageningen University and Research, Droevendaalsesteeg 3, 6708 PB Wageningen, The Netherlands

* Corresponding author: naresh.kumar@wur.nl; walter.schenkeveld@wur.nl

Supporting Information

15 pages
2 text section
9 figures
3 equations
1 table

Table of Content

Table of Figures S3

Vivianite crystal structure S5

Equilibrium concentration of dissolved P of vivianite in comparison to other phosphate minerals . S6

XRD diffractograms S6

Solubility product of vivianite in comparison to previous studies S7

Results and Discussion: Flow-through dissolution experiments S7

Oxidation degree of vivianite suspensions S10

SEM images of vivianite before and after dissolution experiments S10

Temperature dependent dissolution kinetics S12

Solubility product as function of molar surface area S13

Optimization of vivianite stoichiometry with respect to K_{sp} S13

References S15

Table of Figures

Figure S1: Crystal structure of vivianite viewed in a) standard orientation, b) a-b plane illustrating single $Fe(II)_A$ and double, edge sharing $Fe(II)_B$ octahedra, c) b-c plane and d) c-a plane. In brown, Fe octahedra, and purple: PO_4 tetrahedra. Red: oxygen atoms and whitish: Hydrogen atoms. Hydrogen bridge bonds were omitted for clarity. The unit cell is indicated by black lines, and the (010)-plane, responsible for a.o. the perfect cleavage of vivianite, is illustrated in pink. The crystal structure was drawn using the VESTA software[1] based on the crystallographic information by Capitelli, et al. [2]. S5

Figure S2: Modelled total dissolved equilibrium concentration of PO_4 [M] with various phosphate minerals over pH ($CaHPO_4$: calciumhydrogenphosphate) after stoichiometric dissolution. Modelled solutions contain 10 mM NaCl as background electrolyte and infinite amounts of the respective solid phase. S6

Figure S3: X-ray diffractogram of vivianite before and after dissolution experiments at different pH values (top-down: pH 9-6, initial vivianite; for pH 5, no solid material could be recovered due to high dissolution). Grey diffractogram shows vivianite reference pattern (Inorganic Crystal Structure Database (ICSD), #423390 [2]) Grey dotted vertical lines indicate main vivianite peaks......................S6

Figure S4: Negative decadic logarithm of the solubility products (pK_{sp}, Eq 1) of vivianite as a function of pH, as determined in multiple studies. Full black circles represent the values determined in this study. Weakly indicated circles represent non-valid data for solubility at pH 5 and 9 (see discussion). Other date points were taken from literature for comparison. (Data Singer 1972, estimated from graph).....S7

Figure S5: CFSTR experiments with initially 0.1 g vivianite (~200 µmoles) at pH 6.0 (IS=10 mM) under anoxic conditions. a) Dissolved Fe and P concentration over time (error bars indicate deviation between duplicates). Black dotted line denotes the respective flow rate. b) Initial surface area normalized dissolution rate of vivianite (Diss. Rate) over time (black circles); dissolution rate corrected for decreasing surface area assuming spherical particles (Diss. Rate (Dec. SA), black-striped triangles) based on the observed cumulative dissolution (CumDiss, grey dotted line).......................................S8

Figure S6: Oxidation degree (Fe(III)/Fe(tot)) of vivianite suspension (200 µM) during a dissolution experiment over an environmentally relevant pH range (5-9) under anoxic conditions in buffered solution (IS = 10 mM). Error bars indicate deviations between duplicates. S10

Figure S7: Macro- and microscopic images of synthesized vivianite before and after experiments. a) macroscopic image of dried synthesized vivianite powder under anoxic conditions after grinding; b) SEM image of dried synthesized vivianite powder; c) SEM image of a sample taken from a 100 mM vivianite stock suspension; d-f) SEM images of vivianite after 50 hrs dissolution experiment at pH 7, 8 and 9, respectively. g-k) SEM images of remaining vivianite aggregates after ~30 days CFSTR experiment before (g-i) and after sonication (j. k). Respective magnifications are indicated in each image title. S11

Figure S8: Dissolution of vivianite (200 μM) over the temperature range of 5-75°C under anoxic conditions in buffered solution (IS=10 mM). (a) dissolved PO_4 and (b) Fe concentration over time. Error bars indicate deviations between duplicates. Prefixed outtakes illustrate initial dissolution for a better readability. The magnified area is indicated in the figure by the black frame. S12
Figure S9: Solubility constant as a function of molar surface area, calculated according to Eq 15, with γ: 0.31 J m^{-2}. Filled black circle: measured molar surface area of the synthesized vivianite. Filled black square: hydrated ions (vivianite completely dissolved). S13

Vivianite crystal structure

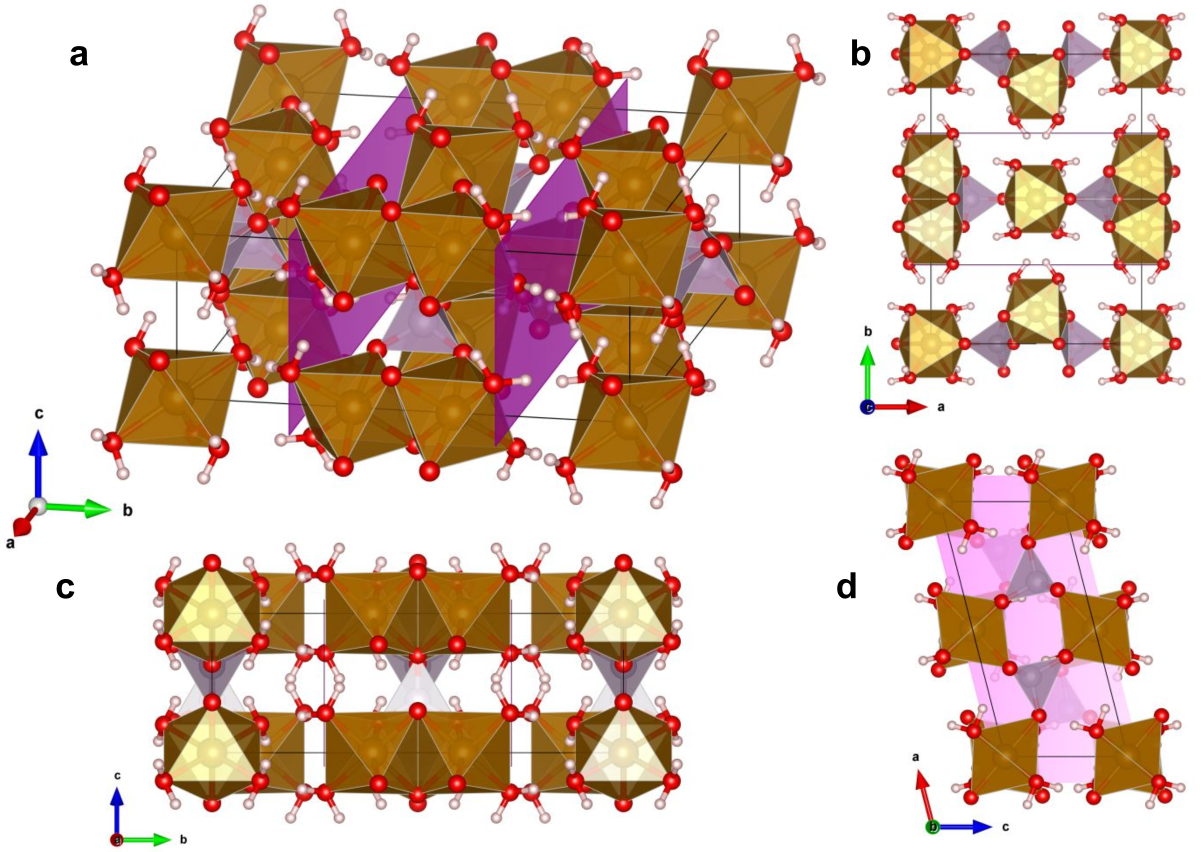

Figure S1: Crystal structure of vivianite viewed in a) standard orientation, b) a-b plane illustrating single $Fe(II)_A$ and double, edge sharing $Fe(II)_B$ octahedra, c) b-c plane and d) c-a plane. In brown, Fe octahedra, and purple: PO_4 tetrahedra. Red: oxygen atoms and whitish: Hydrogen atoms. Hydrogen bridge bonds were omitted for clarity. The unit cell is indicated by black lines, and the (010)-plane, responsible for a.o. the perfect cleavage of vivianite, is illustrated in pink. The crystal structure was drawn using the VESTA software[1] based on the crystallographic information by Capitelli, et al. [2].

Equilibrium concentration of dissolved P of vivianite in comparison to other phosphate minerals

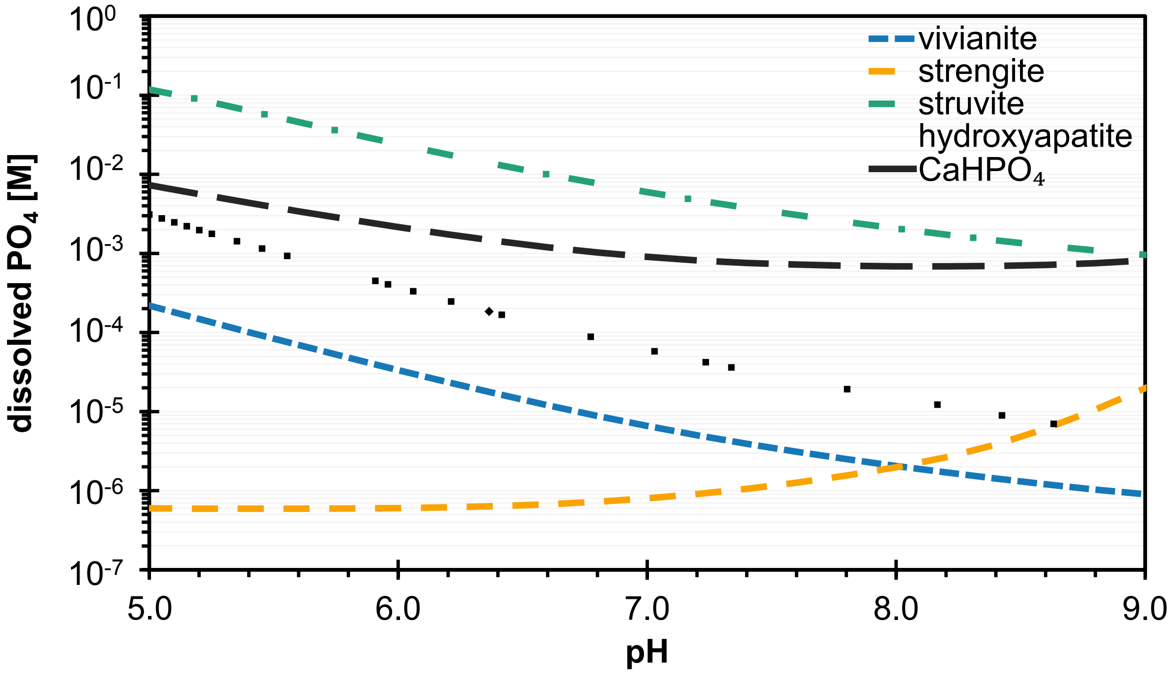

Figure S2: Modelled total dissolved equilibrium concentration of PO_4 [M] with various phosphate minerals over pH ($CaHPO_4$: calcium hydrogen phosphate) after stoichiometric dissolution. Modelled solutions contain 10 mM NaCl as background electrolyte and infinite amounts of the respective solid phase.

XRD diffractograms

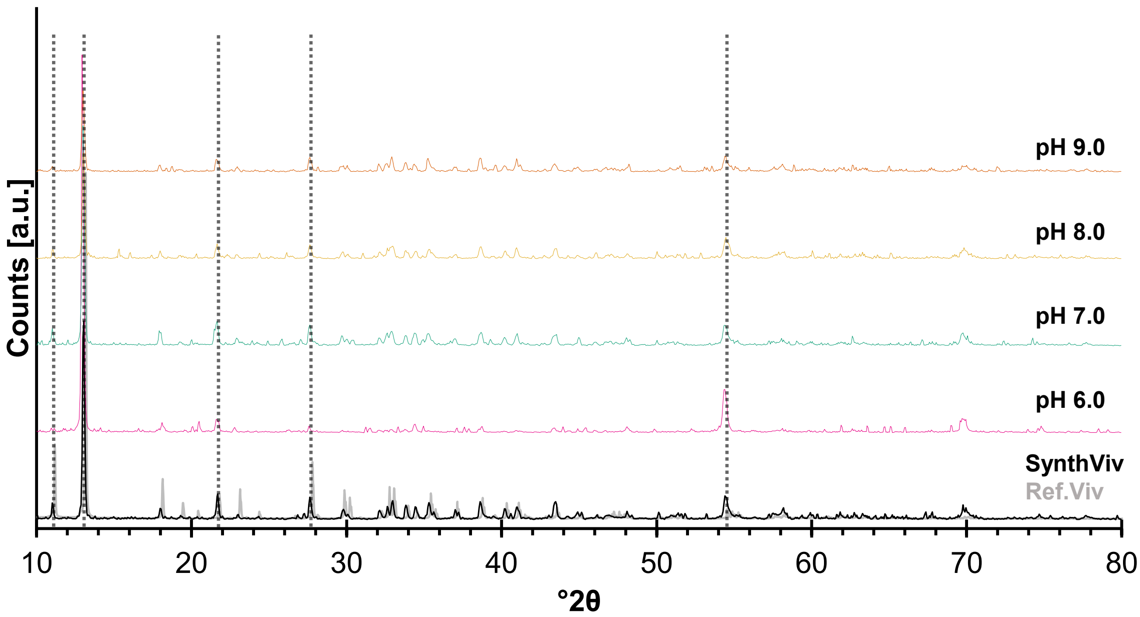

Figure S3: X-ray diffractogram of vivianite before and after dissolution experiments at different pH values (top-down: pH 9-6, initial vivianite; for pH 5, no solid material could be recovered due to high dissolution). Grey diffractogram shows vivianite

reference pattern (Inorganic Crystal Structure Database (ICSD), #423390 [2]) Grey dotted vertical lines indicate main vivianite peaks

Solubility product of vivianite in comparison to previous studies

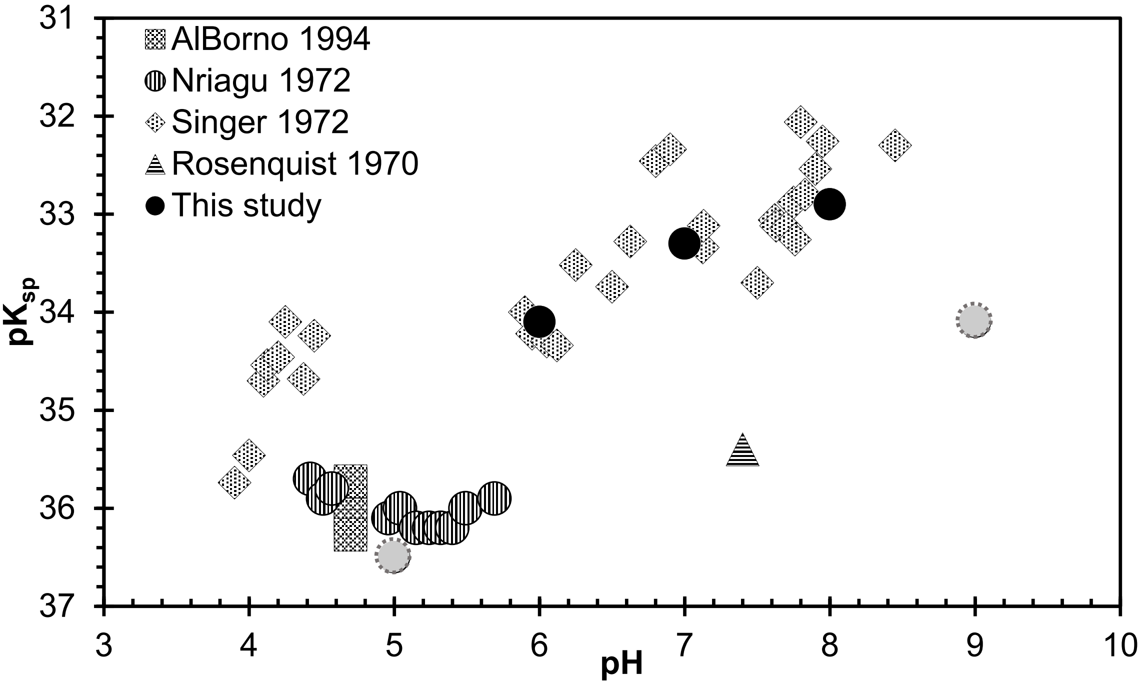

Figure S4: Negative decadic logarithm of the solubility products (pK_{sp}, Eq 1) of vivianite as a function of pH, as determined in multiple studies. Full black circles represent the values determined in this study. Weakly indicated circles represent non-valid data for solubility at pH 5 and 9 (see discussion). Other date points were taken from literature for comparison. (Data Singer 1972, estimated from graph)

Results and Discussion: Flow-through dissolution experiments

For studying dissolution kinetics, CFSTR experiments are in general preferred over batch experiments, since dissolution products do not accumulate in the reactor. R_{exp} in [mol m^{-2} h^{-1}] for a CFSTR experiment can be described by Eq S1:

$$\frac{V}{SSA \cdot m} \cdot \frac{dC}{dt} = (C_{in} - C_{out}) \cdot \frac{Q}{SSA \cdot m} + R_{exp} \quad \text{(S1)}$$

Where V is the reactor volume [L], C_{in} and C_{out} are the total dissolved analyte concentration in the influent and effluent (mol L^{-1}), respectively, Q is the flow rate (L h^{-1}), SSA is the specific surface area [m^2 g^{-1}] and m is the mass of added solid in [g]. At steady state (dC/dt = 0), and if $C_{in} = 0$, Eq S1 can be simplified and easily rearranged for R_{exp}; Eq S2:

$$R_{exp} = \frac{C_{out} \cdot Q}{SSA \cdot m} \quad \text{(S2)}$$

If the concentration of reaction products is kept very low, an influence of solution saturation on the R_{exp} according to Eq S1 is minimized and steady state dissolution under far-from-equilibrium conditions can be approximated ($R_{exp} \approx R_{diss}$). However, this is only true if the residence time of the solute in the reactor is substantially shorter than the time until solution saturation is approached. This can be tested by altering the residence time, since R_{diss} must be independent of the residence time under far-from equilibrium conditions according to Eq 6 (main text).

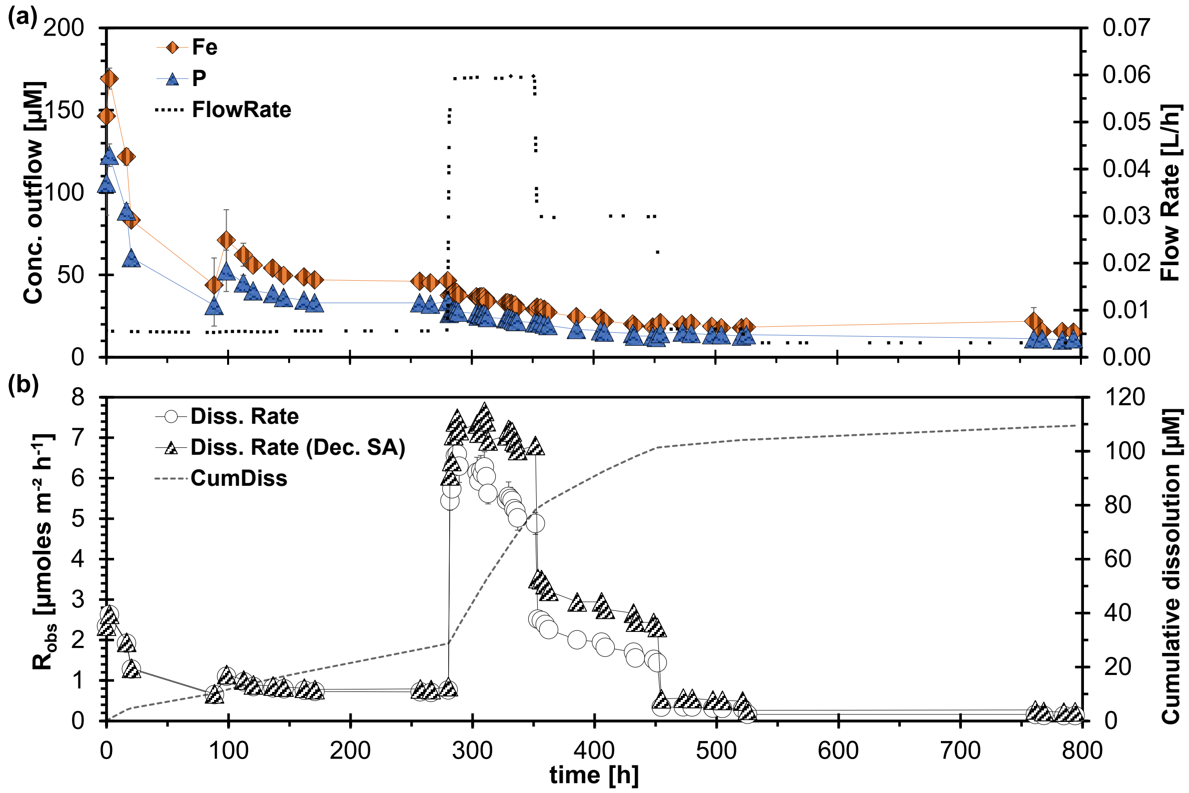

Figure S5: CFSTR experiments with initially 0.1 g vivianite (~200 µmoles) at pH 6.0 (IS=10 mM) under anoxic conditions. a) Dissolved Fe and P concentration over time (error bars indicate deviation between duplicates). Black dotted line denotes the respective flow rate. b) Initial surface area normalized dissolution rate of vivianite (Diss. Rate) over time (black circles); dissolution rate corrected for decreasing surface area assuming spherical particles (Diss. Rate (Dec. SA), black-striped triangles) based on the observed cumulative dissolution (CumDiss, grey dotted line).

CFSTR experiments were performed. Figure S5 b shows the cumulative dissolution of 0.1 g vivianite as well as the surface area normalized dissolution rates over ~33 days of a CFSTR run with and without decreasing surface area correction, assuming spherical particles, at changing pumping rates. Each pumping rate was maintained until at least 15 times 90 mL reactor volumes were exchanged. Throughout the experiment, measured Fe and P concentrations in the outflow solution had the stoichiometric ratio of the mineral, implying that formation of secondary phases, e.g. due to Fe oxidation, could be excluded, and that anoxic vivianite dissolution was the only reaction to be considered. Therefore, dissolution rates could be derived (Eq S2) using the Fe and P outflow concentrations, which were averaged and normalized according to the vivianite stoichiometry.

After a fast-initial dissolution during the first ~100 h (~6 reactor volumes, dissolution of ~6 % of the initial material), dissolved Fe and P concentration approached an apparent steady-state for the initial flow rate of 0.1 mL min^{-1}. An increase in flow rate to 2 mL min^{-1} after 280 h led to a strong increase in dissolution rate, since outflow concentrations remained almost unaltered. The change in dissolution rate immediately following the change in pumping rate indicates a very fast dissolution rate, where Fe and P rapidly approach equilibrium concentrations suggesting a thermodynamically controlled system. However, stop-flow experiments (not shown) showed that the concentrations further increased at no-flow intervals, implying saturation was not completely reached. This is also evident in Figure S5 at 100 h, where the pump accidentally stopped for a few minutes. A stepwise reduction in flow rate after 350 h, lead to a decrease in the observed dissolution rate again. Obviously a steady-state dissolution rate was not reached during the experiment since R_{exp} followed changes in pumping rate. Moreover, when after ~450 h the flow rate was back to the initial rate of 0.1 mL min^{-1}, the calculated dissolution rate was lower than during the first interval and also slightly decreased further over time. SEM images of the solids remaining at the end of the experiment confirmed strong agglomeration of smaller vivianite particles (Figure S7 g-i), which might explain the slight decrease in dissolution rate over a longer time period. The vivianite agglomerates could be easily broken down by sonication (Figure S7 j-k).

Dissolution of highly soluble minerals has a greater likelihood of becoming transport-limited as compared to low-solubility minerals, which are likely to be interface-limited.[3] Consequently, we also examined if vivianite dissolution was transport controlled in our experiments, by setting up reactors with different stirring speeds (250 and 1000 rpm). However, no differences were observed, suggesting that dissolution was not diffusion controlled.

Due to the relatively long duration of the experiment and the high dissolution rates, a substantial fraction of the vivianite dissolved (> 50 %, ~100 µmole); see Figure S5 b dotted line: cumulative dissolution), and the effect of decreasing surface area on the dissolution rate could not be neglected. Therefore, a mass-based correction according to Tang *et. al.* [4] (Eq 9, main text) was used to recalculate dissolution rates (Figure S5 b striped triangles). Nevertheless, R_{exp} became more constant for each pumping rate, but not between the different pumping rates. Therefore, it can be concluded, that no steady-state dissolution could be reached with CFSTR setup, since R_{exp} was mainly governed by the pumping rate and thus solution saturation. The potential effect from agglomeration on the SSA however, is not accounted for in the mass-based correction.

Oxidation degree of vivianite suspensions

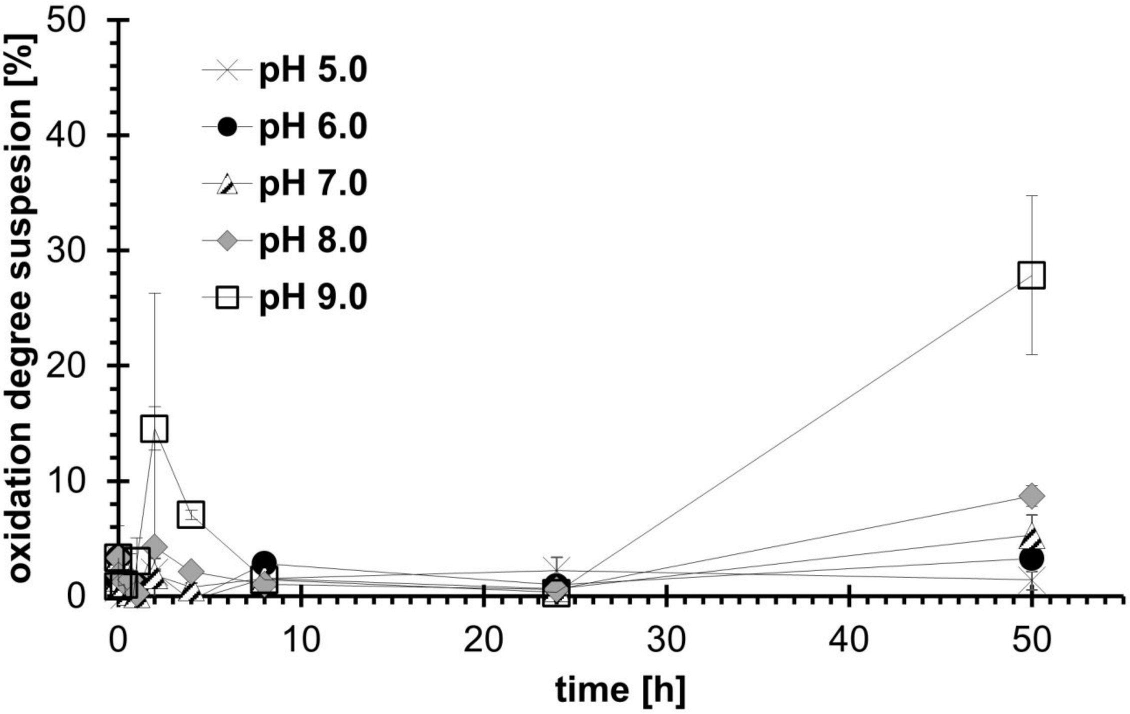

Figure S6: Oxidation degree (Fe(III)/Fe(tot)) of vivianite suspension (200 µM) during a dissolution experiment over an environmentally relevant pH range (5-9) under anoxic conditions in buffered solution (IS = 10 mM). Error bars indicate deviations between duplicates.

SEM images of vivianite before and after dissolution experiments

a) SynthViv powder	b) SynthViv powder 10000x	c) SynthViv sus. 4000x
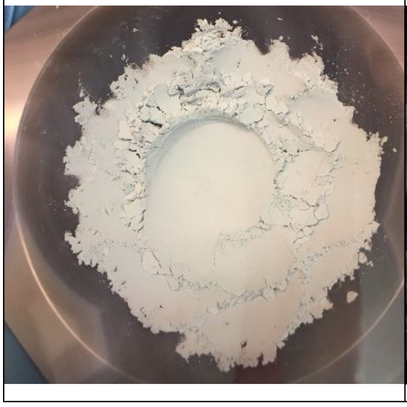	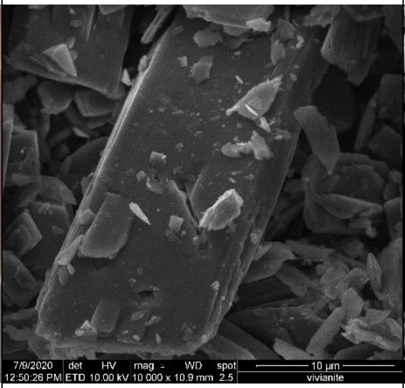	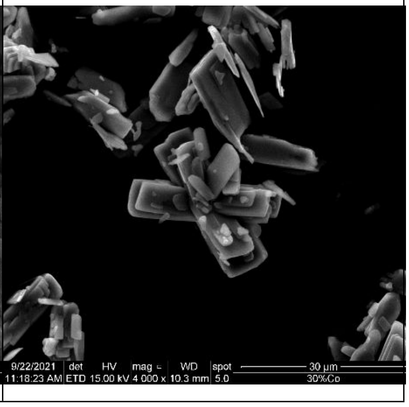

d) pH 7 2500x	e) pH 8 2500x	f) pH 9 2500x
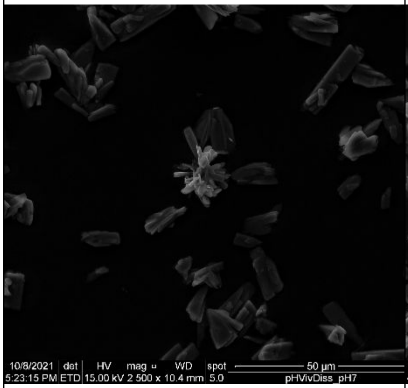	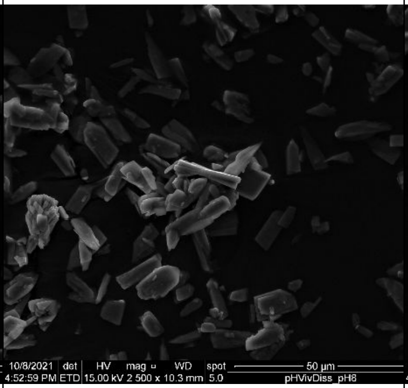	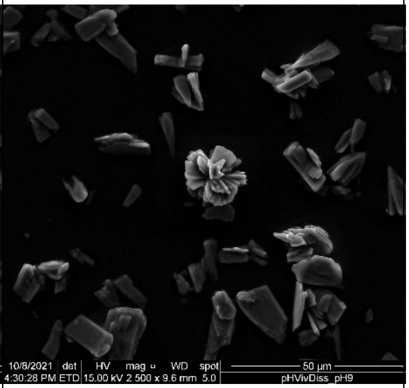
g) CFSTR 500x	h) CFSTR 2000x	i) CFSTR 4000x
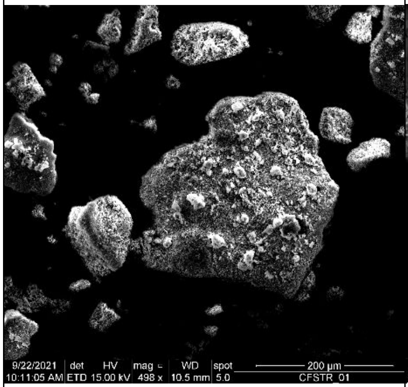	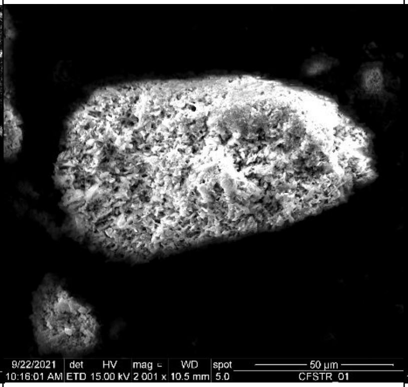	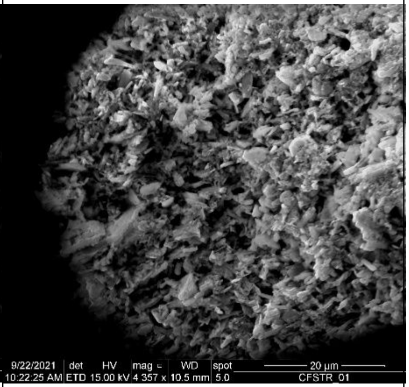
j) CFSTR sonicate 2500x	k) CFSTR sonicate 10000x	
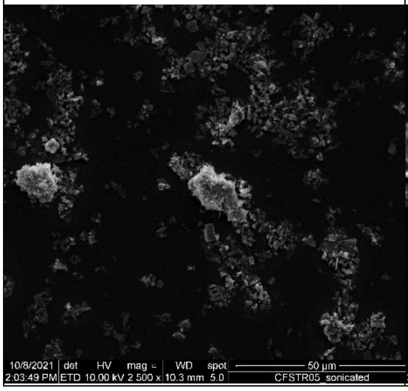	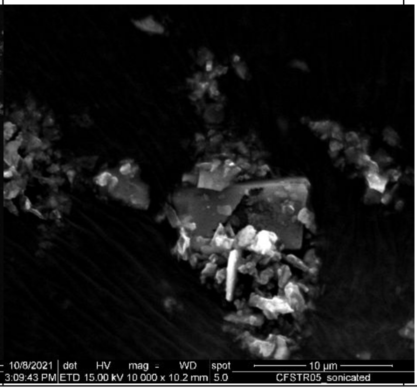	

Figure S7: Macro- and microscopic images of synthesized vivianite before and after experiments. a) macroscopic image of dried synthesized vivianite powder under anoxic conditions after grinding; b) SEM image of dried synthesized vivianite powder; c) SEM image of a sample taken from a 100 mM vivianite stock suspension; d-f) SEM images of vivianite after 50 hrs dissolution experiment at pH 7, 8 and 9, respectively. g-k) SEM images of remaining vivianite aggregates after ~30 days CFSTR experiment before (g-i) and after sonication (j. k). Respective magnifications are indicated in each image title.

Temperature dependent dissolution kinetics

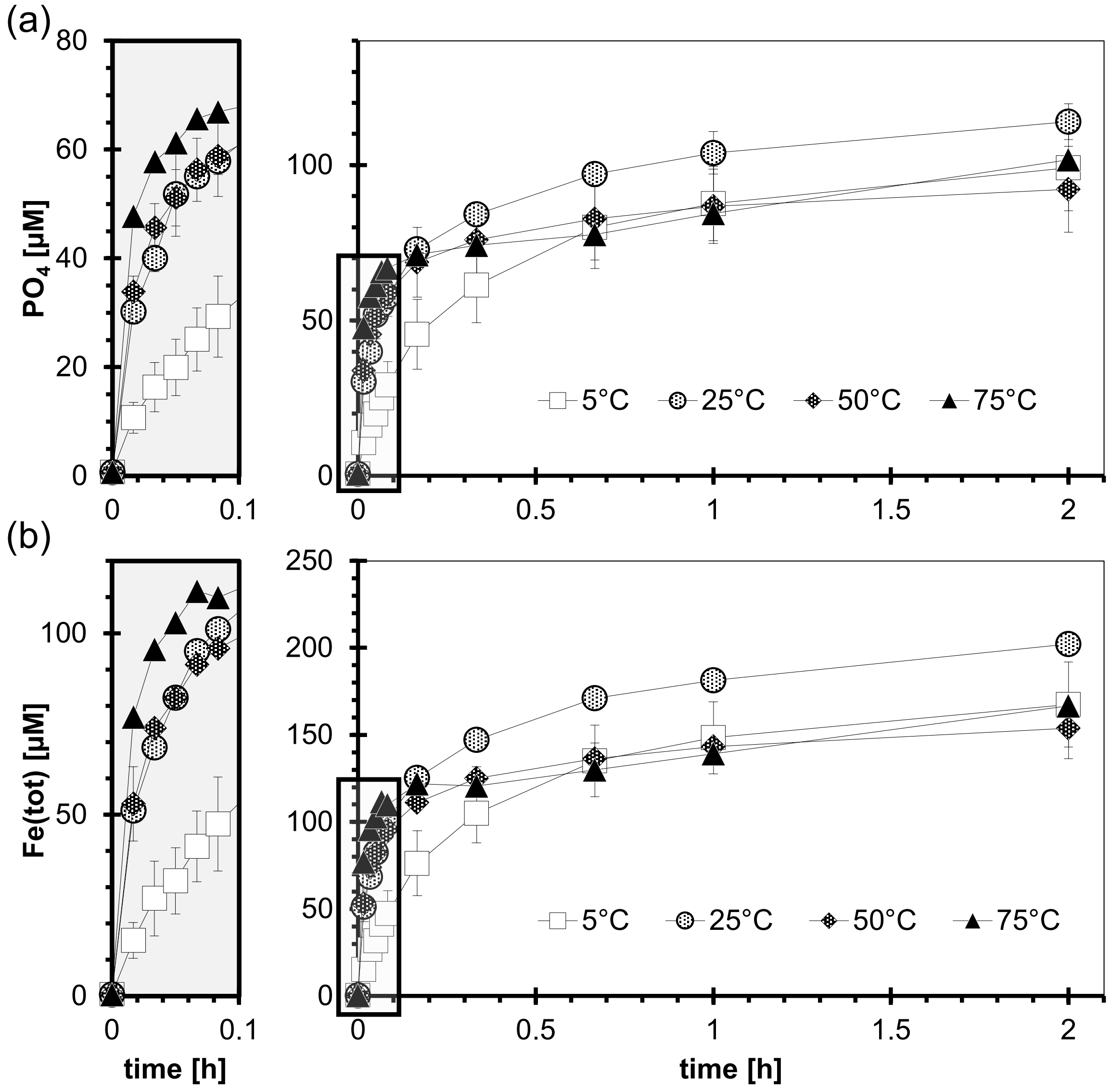

Figure S8: Dissolution of vivianite (200 µM) over the temperature range of 5-75°C under anoxic conditions in buffered solution (IS=10 mM). (a) dissolved PO_4 and (b) Fe concentration over time. Error bars indicate deviations between duplicates. Prefixed outtakes illustrate initial dissolution for a better readability. The magnified area is indicated in the figure by the black frame.

Solubility product as function of molar surface area

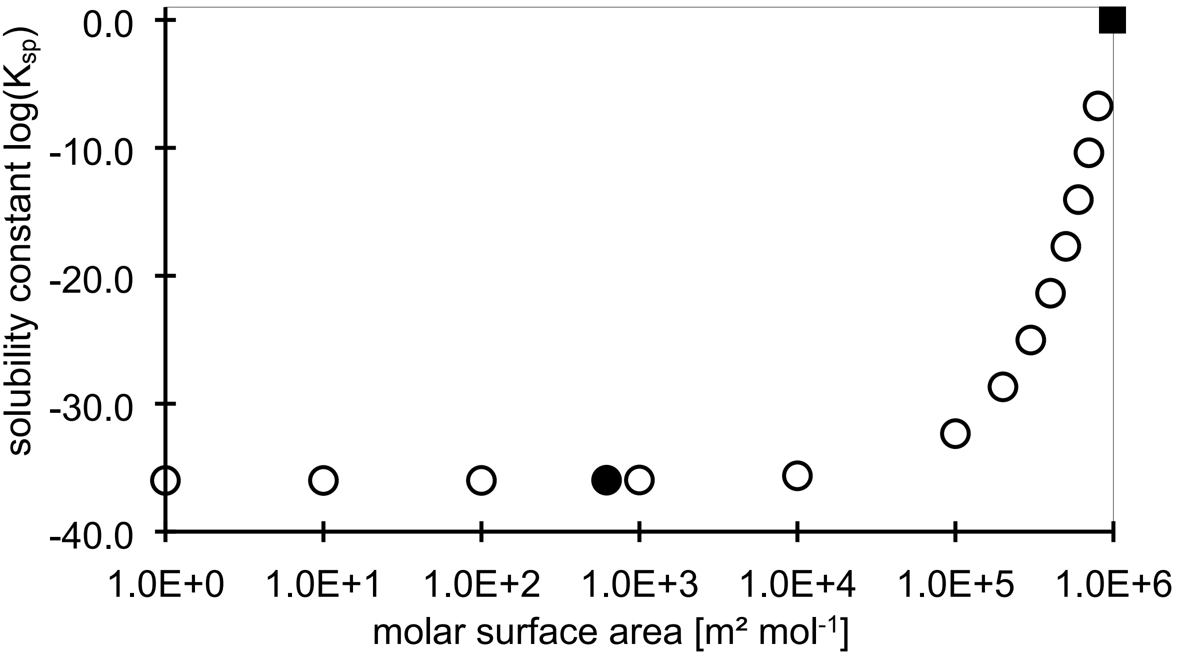

Figure S9: Solubility constant as a function of molar surface area, calculated according to Eq 15, with γ: 0.31 J m^{-2}. Filled black circle: measured molar surface area of the synthesized vivianite. Filled black square: hydrated ions (vivianite completely dissolved).

Optimization of vivianite stoichiometry with respect to K_{sp}

The experimentally determined pH value, dissolved Fe(II) and dissolved P concentration was used to model species distribution and the according activities of $\{Fe^{2+}\}$ and $\{PO_4^{3-}\}$ using Visual Minteq 3.1 and its associated thermodynamic database (thermo.vdb). MS office Excel Add-In Solver was used to minimize the sum of the relative differences between the experimental activities and the calculated activities (Table S1), which were calculated by rearranging the solubility product of vivianite including $\{H^+\}$: Equation S3:

$$K_{sp_{pH6-8}} = \{Fe^{2+}\}^b \cdot \{PO_4^{3-}\}^2 \cdot \{H^+\}^a \quad \text{(S3)}$$

The stoichiometric exponents *a* and *b* were used as variables and were only restricted by charge balance expressed as: $a + 2b = 6$. The data for pH 5 and 9 were omitted due to complete dissolution and non-stoichiometric dissolution, respectively (see main text).

Table S1: optimization of vivianite stoichiometry to compensate for apparent increasing solubility product with increasing pH. Experimental and calculated species distribution over pH and their relative differences. The results for pH 5 and 9 were omitted for calculations.

		pH	5	6	7	8	9
experimental		{H+}	1.00E-05	1.00E-06	1.00E-07	1.00E-08	1.00E-09
		{Fe2+}	3.41E-04	2.17E-04	5.25E-05	1.39E-05	1.55E-06

		{PO43-}	1.36E-13	3.84E-12	4.55E-11	4.73E-10	5.88E-09
		pH	5	6	7	8	9
calculated		{H+}	3.11E-01	1.00E-06	1.00E-07	1.00E-08	1.23E-07
		{Fe2+}	1.68E-03	2.17E-04	5.25E-05	1.39E-05	3.25E-06
		{PO43-}	1.25E-12	3.84E-12	4.55E-11	4.73E-10	1.65E-08
		pH	5	6	7	8	9
relative difference (experimental – calculated)		{H+}	3.11E+04	8.24E-06	3.22E-06	1.55E-05	1.22E+02
		{Fe2+}	3.92E+00	1.27E-06	4.96E-07	2.38E-06	1.10E+00
		{PO43-}	8.20E+00	1.77E-06	6.91E-07	3.32E-06	1.81E+00
Minimize sum of relative differences				3.68E-05			

References

1 Momma, K. & Izumi, F. VESTA 3 for three-dimensional visualization of crystal, volumetric and morphology data. *Journal of Applied Crystallography* **44**, 1272-1276 (2011). https://doi.org:http://doi.org/doi:10.1107/S0021889811038970

2 Capitelli, F., Chita, G., Ghiara Maria, R. & Rossi, M. in *Zeitschrift für Kristallographie Crystalline Materials* Vol. 227 92 (2012).

3 Brantley, S. L. in *Kinetics of Water-Rock Interaction* (eds Susan L. Brantley, James D. Kubicki, & Art F. White) 151-210 (Springer New York, 2008).

4 Tang, R., Wu, W., Haas, M. & Nancollas, G. H. Kinetics of Dissolution of β-Tricalcium Phosphate. *Langmuir* **17**, 3480-3485 (2001). https://doi.org:https://doi.org/10.1021/la001730n
